# Development of a Mitochondrial Myopathy-Composite Assessment Tool

**Published:** 2021-08-30

**Authors:** Jean Flickinger, Jiaxin Fan, Amanda Wellik, Rebecca Ganetzky, Amy Goldstein, Colleen C. Muraresku, Allan M. Glanzman, Elizabeth Ballance, Kristin Leonhardt, Elizabeth M. McCormick, Brianna Soreth, Sara Nguyen, Jennifer Gornish, Ibrahim George-Sankoh, James Peterson, Laura E. MacMullen, Shailee Vishnubhatt, Michael McBride, Richard Haas, Marni J. Falk, Rui Xiao, Zarazuela Zolkipli-Cunningham

**Affiliations:** 1 Mitochondrial Medicine Frontier Program, Division of Human Genetics, Department of Pediatrics, Children's Hospital of Philadelphia, Philadelphia, PA 19104, USA; 2 Department of Physical Therapy, Children's Hospital of Philadelphia, Philadelphia, PA, USA; 3 Department of Biostatistics, Epidemiology and Informatics, University of Pennsylvania Perelman School of Medicine, Philadelphia, PA, USA; 4 Department of Pediatrics, University of Pennsylvania Perelman School of Medicine, Philadelphia, PA, USA; 5 Cardiovascular Exercise Physiology Laboratory, Division of Cardiology, Children's Hospital of Philadelphia, Philadelphia, PA, USA; 6 Metabolic and Mitochondrial Disease Center, La Jolla, CA, USA; 7 Department of Neurosciences, University of California San Diego School of Medicine, La Jolla, CA, USA

**Keywords:** Mitochondrial myopathy, Muscle weakness, Muscle fatigue, Exercise intolerance, Outcome measures, Composite measure

## Abstract

**Background:**

‘Mitochondrial Myopathy’ (MM) refers to genetically-confirmed Primary Mitochondrial Disease (PMD) that predominantly impairs skeletal muscle function. Validated outcome measures encompassing core MM domains of muscle weakness, muscle fatigue, imbalance, impaired dexterity, and exercise intolerance do not exist. The goal of this study was to validate clinically-meaningful, quantitative outcome measures specific to MM.

**Methods:**

This was a single centre study. Objective measures evaluated included hand-held dynamometry, balance assessments, Nine Hole Peg Test (9HPT), Functional Dexterity Test (FDT), 30 second Sit to Stand (30s STS), and 6-minute walk test (6MWT). Results were assessed as *z*-scores, with < −2 standard deviations considered abnormal. Performance relative to the North Star Ambulatory Assessment (NSAA) of functional mobility was assessed by Pearson’s correlation.

**Results:**

In genetically-confirmed MM participants [*n* = 59, mean age 21.6 ± 13.9 (range 7 – 64.6 years), 44.1% male], with nuclear gene aetiologies, *n* = 18/59, or mitochondrial (mtDNA) aetiologies, *n* = 41/59, dynamometry measurements demonstrated both proximal [dominant elbow flexion (−2.6 ± 2.1, mean *z*-score ± standard deviation, SD), hip flexion (−2.5 ± 2.3), and knee flexion (−2.8 ± 1.3)] and distal muscle weakness [wrist extension (−3.4 ± 1.7), palmar pinch (−2.5 ± 2.8), and ankle dorsiflexion (−2.4 ± 2.5)]. Balance [Tandem Stance (TS) Eyes Open (−3.2 ± 8.8, *n* = 53) and TS Eyes Closed (−2.6 ± 2.7, *n* = 52)] and dexterity [FDT (−5.9 ± 6.0, *n* = 44) and 9HPT (−8.3 ± 11.2, *n* = 53)] assessments also revealed impairment. Exercise intolerance was confirmed by strength-based 30s STS test (−2.0 ± 0.8, *n* = 38) and mobility-based 6MWT mean *z*-score (−2.9 ± 1.3, *n* = 46) with significant decline in minute distances (slope −0.9, *p* = 0.03, *n* = 46). Muscle fatigue was quantified by dynamometry repetitions with strength decrement noted between first and sixth repetitions at dominant elbow flexors (−14.7 ± 2.2%, mean ± standard error, SEM, *n* = 21). All assessments were incorporated in the MM-Composite Assessment Tool (MM-COAST). MM-COAST composite score for MM participants was 1.3± 0.1(*n* = 53) with a higher score indicating greater MM disease severity, and correlated to NSAA (*r* = 0.64, *p* < 0.0001, *n* = 52) to indicate clinical meaning. Test–retest reliability of MM-COAST assessments in an MM subset (*n* = 14) revealed an intraclass correlation coefficient (ICC) of 0.81 (95% confidence interval: 0.59–0.92) indicating good reliability.

**Conclusions:**

We have developed and successfully validated a MM-specific Composite Assessment Tool to quantify the key domains of MM, shown to be abnormal in a Definite MM cohort. MM-COAST may hold particular utility as a meaningful outcome measure in future MM intervention trials.

## Background

Primary mitochondrial disease (PMD) is a clinically heterogeneous group of several hundred gene disorders,^1^ caused by pathogenic variants in either nuclear DNA (nDNA) or mitochondrial DNA (mtDNA), which collectively affect at least one in 4,300 people across all ages.^2^ Defects of oxidative phosphorylation (OXPHOS) typically affect high-energy organs such as skeletal muscle and the brain.^3^ ‘Mitochondrial Myopathy’ (MM) refers to a subset of PMD that predominantly, although not exclusively, impairs skeletal muscle function.^3,4^ Currently, no FDA approved therapies exist for MM.^5^ A major barrier to establishing approved therapies is a lack of MM specific outcome measures. While muscle weakness is the predominant symptom in other genetic neuromuscular disorders, such as Duchenne Muscular Dystrophy (DMD) or Spinal Muscular Atrophy (SMA), exercise intolerance and muscle fatigue often exist in the absence of overt muscle weakness in MM. Therefore, an assessment that focuses solely on muscle strength would not fully reflect MM disease severity. The Newcastle Mitochondrial Disease Adult Scale (NMDAS)^6^ is a semi-quantitative clinical rating scale designed to reflect the multi-system burden of mitochondrial disease and has been utilized in clinical trials to measure change over short treatment intervals (for which it had not been validated) with negative results.^7,8^ The NMDAS does not include objective assessments. The 6-minute walk test (6MWT)^7^ and cardiopulmonary exercise testing (CPET)^8^ are the only objective measures implemented in recent MM clinical trials. The clinical meaning of the total distance walked in 6MWT is unclear.^7^ CPET is a valuable, non-invasive method of measuring oxygen consumption in mitochondrial disease patients. In our clinical experience, individuals who are not able to follow commands, have significant ataxia or extrapyramidal movements, have height <135 cm, and are young children, are not able to reliably complete CPET testing, limiting its broad utility across the age spectrum in MM. Thus, a critical need exists to validate MM-specific objective Foutcome measures that quantify clinically meaningful patient-reported key domains.^9^

In this study, we defined participants with MM as having genetically confirmed PMD due to pathogenic or likely pathogenic variants in either nuclear DNA or mtDNA that impair mitochondrial structure and/or function, with clinical symptoms of muscle weakness, exercise intolerance, muscle fatigue, and/or imbalance.^9^ The objective of this study was to validate clinically meaningful quantitative measures specific to core domains of MM across all ages in a combined child (<18 years) and adult MM cohort at a single-site Mitochondrial Medicine clinical centre, for implementation in future longitudinal studies to measure disease severity over time and following intervention in a clinical trial.

### Methods

We studied a total of 59 ‘Definite MM’ participants symptomatic of myopathy at time of enrolment (Table 1; Supporting Information, Table S1), as well as 32 ‘Unlikely MM’ participants (as defined by Bernier diagnostic classification)^10^ who had similar symptomatology but were not subsequently confirmed to harbour an MM genetic aetiology on clinical whole exome sequencing and, where possible, muscle biopsy diagnostic testing. We compared objective assessments in participants with ‘Definite’ compared to ‘Unlikely’ MM who were symptomatic of myopathy and evaluated in our Mitochondrial Centre, in order to validate the MM-Composite Assessment Tool (MM-COAST) as a clinically meaningful quantitative assessment tool specific to ’Definite’ MM. We did not compare objective assessments in ’Definite’ MM to healthy volunteers as all results were normalized to normative population data. All participants were enrolled to our Children’s Hospital of Philadelphia (CHOP) Institutional Review Board (IRB) approved studies following individual, or parental-informed consent for participants < 18 years, and participants ≥ 18 years who were unable to provide their own consent [Children’s Hospital of Philadelphia Institutional Review Board #08–6177 (MJF, PI), #16–013364 (ZZC, PI)]. Inclusion criteria included being ≥ 6 years old to ensure reliable completion of assessments. Participants with severe cardiac or pulmonary disease, those with non-MM related symptoms such as injuries or those on medication that would potentially influence ability to complete assessments, were excluded. In this study, 6/59 (10.2%) participants were ‘non-ambulatory’, defined as individuals who are unable to walk ≥ five steps independently.^11^ All ’Definite’ MM participants received standard-of-care management, including mitochondrial vitamin and/or cofactor supplements.^12^

**Figure 1:**
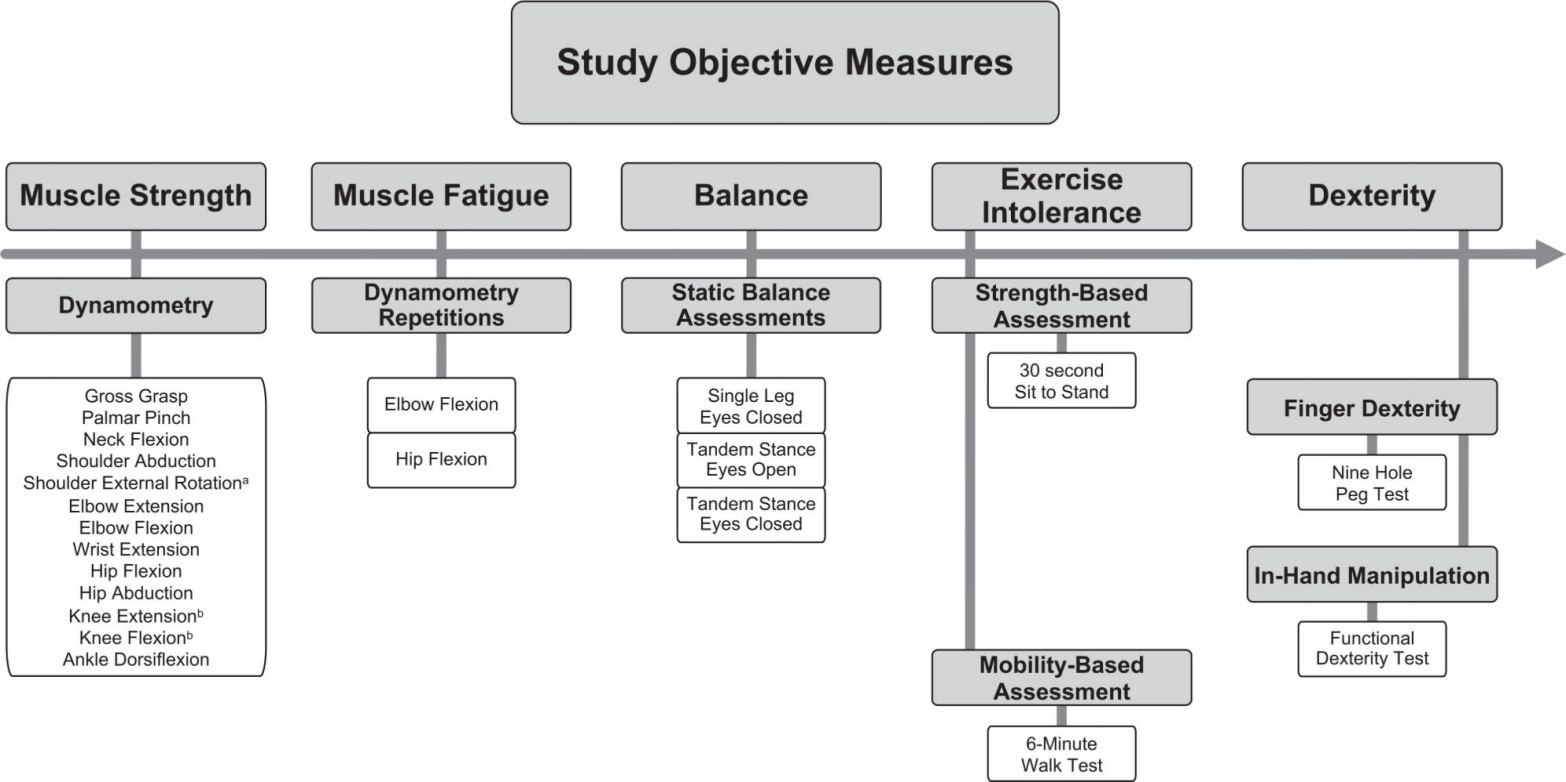
Mitochondrial Myopathy objective measures. Assessments in each of five domains tested and order of assessments is displayed. Measurement of muscle strength using dynamometry was the initial assessment performed, followed by dynamometry repetitions, balance assessments, 30 second Sit to Stand (30s STS), Nine Hole Peg Test (9HPT), Functional Dexterity Test (FDT), and the 6-Minute Walk Test (6MWT). ^a^Muscle group assessed only in adults per protocol. ^b^Muscle group assessed only in children per protocol.

Existing motor performance measures validated in other disorders and considered potentially useful in MM were compiled in a similar way to past efforts,^13,14^ along with MM consensus statements.^3,15^ The relative merits, feasibility, and clinical relevance of existing outcome measures to MM were assessed, which enabled final selection for validation of the following measures (Figure 1, Table S2): hand-held dynamometry for muscle strength^16,17^ including grip and palmar pinch strength,^18,19^ balance testing,^20^ Nine Hole Peg Test (9HPT),^20^ Functional Dexterity Test (FDT),^20^ 30 second Sit to Stand test (30s STS),^20^ and 6MWT total distance^20^ and minute distances.^21^ Each outcome measure was also selected based on the reliability of the assessment protocols and corresponding age-based normative data sets.^16–20^ As we anticipated disease variability across the cohort that is typical of MM, we selected quantitative measures that would avoid floor and/or ceiling effects in MM. All administered assessments adhered to published protocols and were conducted by a skilled physical therapist to ensure reproducibility. We developed a new dynamometry repetitions protocol for assessment of muscle fatigue in hip flexors and elbow flexors, expressed as mean negative percent decrement (Table S3). In order to prevent undue iatrogenic fatigue from prolonged assessments, various sequences of assessments were trialled and adjusted based on MM participant feedback, and the final chronological order was established (Figure 1, Table S2). Existing intra-rater, inter-rater, and test–retest reliability scores for individual assessments are summarized (Table S2). Detailed assessment methods are outlined in Table S3, with relevant clinical observations, advantages, and challenges of each assessment provided in Table S4.

**Figure 2:**
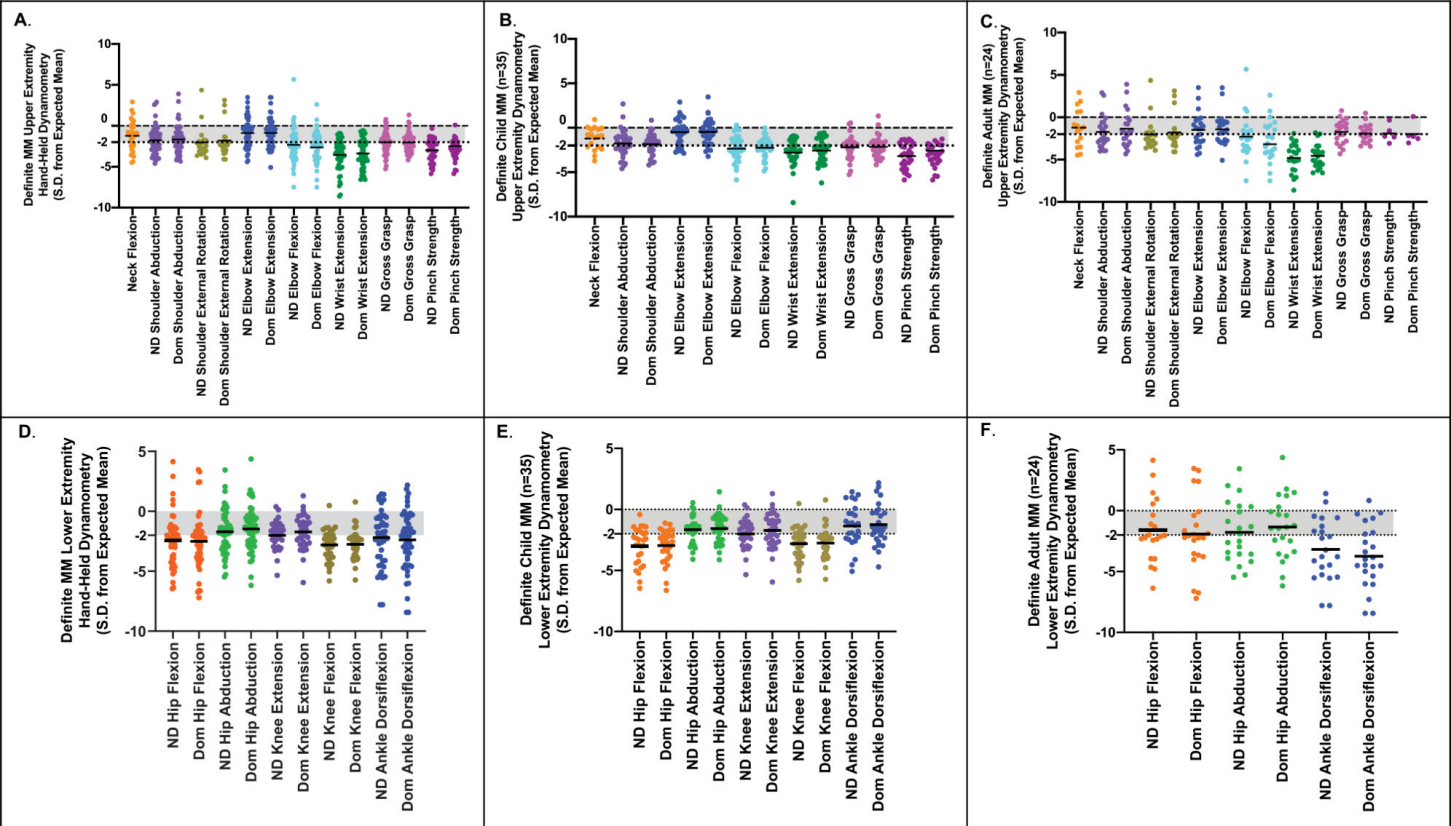
Dynamometry assessments revealed both proximal and distal weakness in Definite MM participants. Data are presented as mean *z*-scores ± standard deviation (SD) on dominant (Dom) and non-dominant (ND) sides. *Z*-scores < −2 is considered abnormal. Shaded area indicates normal *z*-score (0 to −2). (A–C) Dynamometry-measured muscle strength in the upper extremities in the Definite MM group (*n* = 59). (B) Dynamometry measurements in child MM (*n* = 35) indicate muscle weakness in elbow flexion (−2.3 ± 1.1, Dom, −2.4 ± 1.4, ND), wrist extension (−2.6 ± 1.4, Dom, −2.8 ± 1.5, ND), grasp (−2.2 ± 1.2, Dom, −2.2 ± 1.3, ND) and pinch strength (−2.6 ± 3.0, Dom, −3.2 ± 1.4, ND). (C) Dynamometry measurements in adult MM (*n* = 24) indicate muscle weakness in elbow flexion (−3.2 ± 3.0, Dom, −2.3 ± 2.6, ND) and wrist extension (−4.6 ± 1.4, Dom, −4.8 ± 1.8, ND). (D–F) Dynamometry-measured muscle strength in MM lower extremities (*n* = 59). (E) Dynamometry measurements in child MM (*n* = 35) indicate muscle weakness in hip flexion (−3.0 ± 1.4, Dom, −3.0 ± 1.5, ND) and knee flexion (−2.8 ± 1.3, Dom, −2.8 ± 1.3, ND). (F) Dynamometry measurements in adult MM (*n* = 24), indicate muscle weakness in ankle dorsiflexion (−3.8 ± 2.6, Dom, −3.2 ± 2.5, ND).

**Figure 3:**
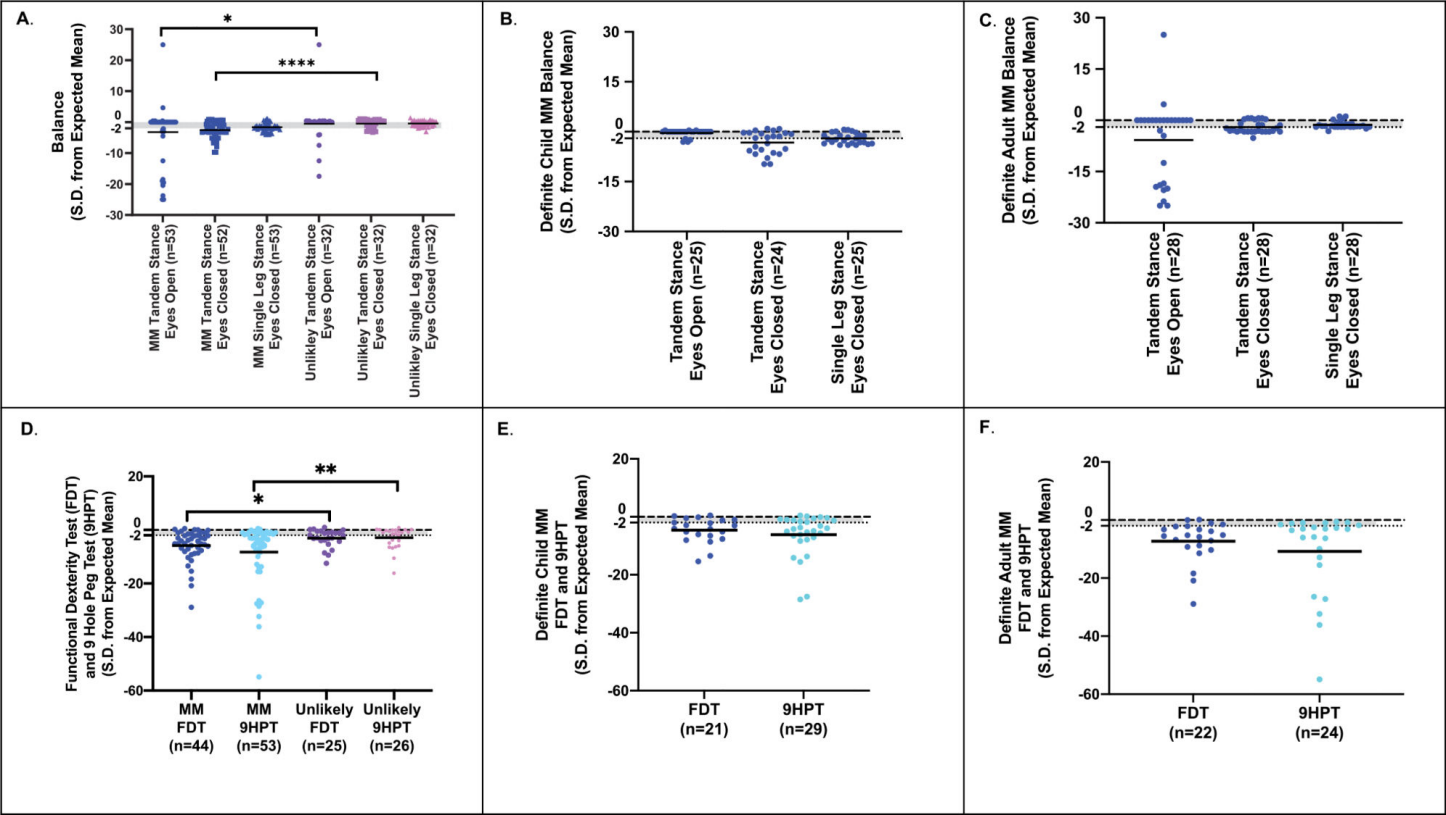
Balance and dexterity assessments revealed deficits in Definite MM compared to Unlikely participants and in child MM vs. adult MM. Data are presented as mean *z*-score ± standard deviation (S.D.). (A) Balance testing was significantly impaired (< −2 S.D.) on Tandem Stance (TS) Eyes Open (*p=0.02, *n* = 53) and Eyes Closed (****p < 0.0001, *n* = 52) in Definite MM compared to the Unlikely MM (*n* = 32) participants. (B) In child MM participants, TS Eyes Closed (−3.2 ± 3.4, *n* = 24) was impaired. (C) In adult MM participants, TS Eyes Open (−5.8 ± 11.6, *n* = 28) was abnormal. (D) FDT (*p = 0.033, *n* = 44) and 9HPT (**p = 0.006, *n* = 53) was significantly more impaired in the Definite MM cohort compared to the Unlikely group (*n* = 25 and *n* = 26, respectively). (E–F) FDT and 9HPT were impaired in child (E) and adult MM (F). There was no significant difference on *t*-test comparison between the FDT and 9HPT *z*-scores in either age group.

**Figure 4:**
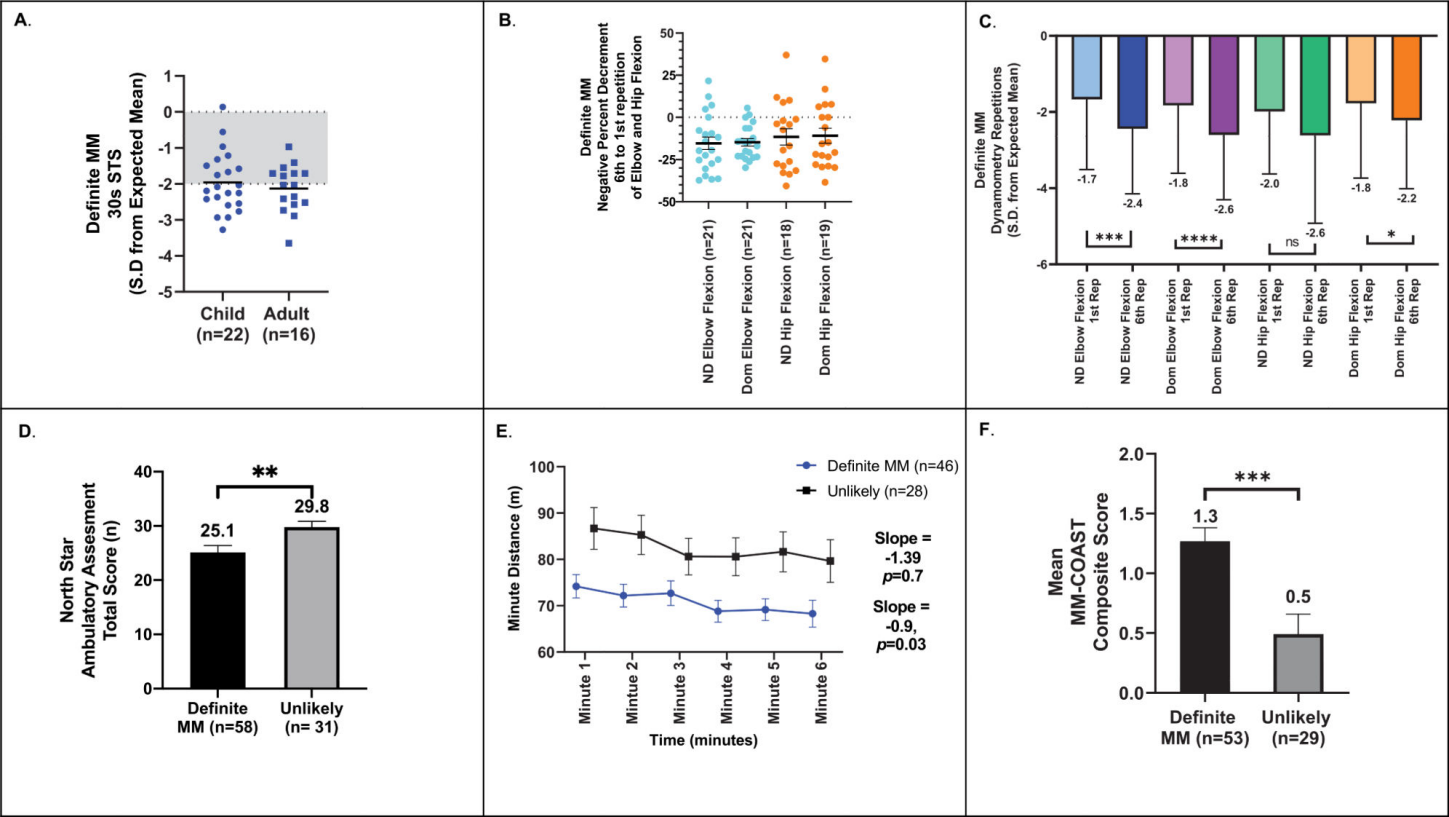
Exercise intolerance, muscle fatigue, and functional assessments revealed deficits in Definite MM participants. (A) 30s STS presented as mean *z*-score ± SD was abnormal (< −2 S.D.) in adult Definite MM (−2.1 ± 0.7, *n* = 16) and borderline abnormal in child Definite MM (−2.0 ± 0.8, *n* = 22). (B) Dynamometry repetitions in Definite MM reveals the mean negative % decrement (mean ± SEM) between the sixth to the first repetition on the non-dominant (ND) and dominant sides at elbow and hip flexion. (C) *T-*test comparison of the *z*-scores ± S.D. between the first and the sixth repetitions, ND (***p = 0.0002) and Dom (****p < 0.0001) sides at elbow flexion, and ND (*p* = 0.13) and Dom (*p = 0.02) sides at hip flexion. (D) The North Star Ambulatory Assessment (NSAA) mean total score was significantly lower (**p = 0.006) in the Definite MM participants (*n* = 58) compared to the Unlikely participants (*n* = 31). (E) Mixed-effects analysis revealed a significant decline in minute distance (m) in Definite MM (slope = −0.9, *p* = 0.03). (F) The mean MM-COAST Composite Score in the Definite MM participants (*n* = 53) was significantly higher than in the Unlikely participants (*n* = 29), ***p = 0.0005.

It is conventional in physical therapy and rehabilitation practice to conduct quantitative assessments and normalize to existing population data. In this study, results were normalized to published normative data^16–20^ that encompassed the full age span relevant to MM, and presented as *z*-scores (equations outlined in Table S3), where < −2 was considered abnormal. A different protocol with its corresponding normative values was selected for children^16,18^ as compared with adults^17,19^ for dynamometry assessments (Figure 2), due to smaller age group clusters spanning only one year in the child normative data set, thus providing more precise normative strength data that accounts for the rapid developmental changes that occur in children.^16^ In comparison, there are broader age groups spanning decades in the adult normative data set.^17^ The same assessment protocol and corresponding normative data was applied to both adult and child participants for dexterity and balance assessments (Figure 3), 30s STS and 6MWT (Figure 4), as age-based normative values across the full age spectrum was available using McKay *et al*.^20^ In this study, hand-held dynamometry assessments were conducted bilaterally (Figures 2 and 4), but only dominant side *z*-scores as determined by writing hand preference were used for correlation analysis. Across 13 distinct muscle groups listed in Figure 1, 11 muscle groups were assessed in adults and 12 in children, in accordance with dynamometry protocols.^16–19^ Shoulder external rotation was measured in adults only, while knee extension and knee flexion were measured in children only.^16–19^

**Figure 5:**
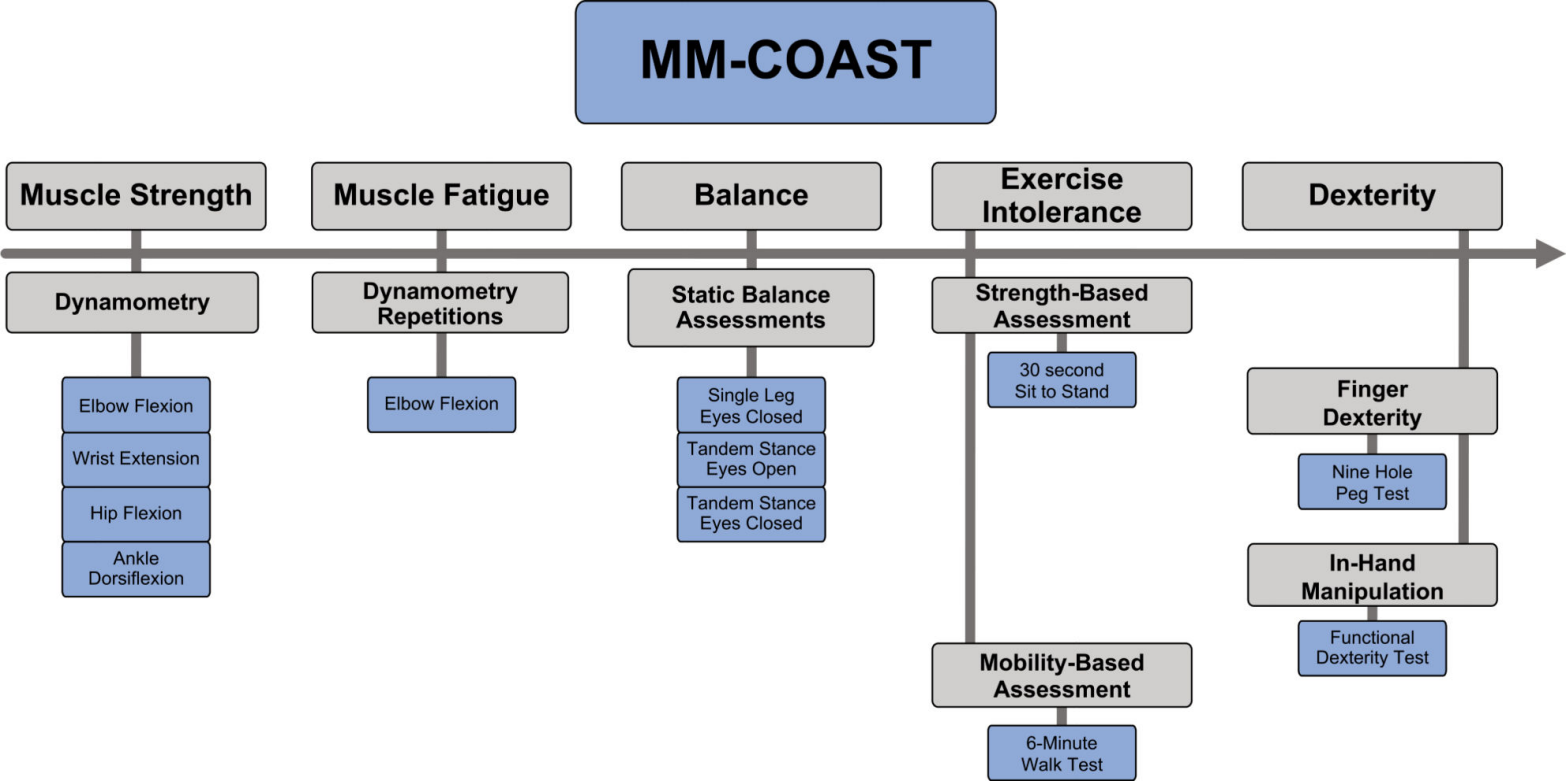
Mitochondrial Myopathy-Composite Assessment Tool (MM-COAST). Assessments selected from study objective measures to be included in the final MM-COAST in each of five domains tested and order of assessments is displayed. Muscle strength assessment of only four muscle groups with dynamometry was the initial assessment performed, followed by dynamometry repetitions of elbow flexion, balance assessments, 30 second Sit to Stand (30s STS), Nine Hole Peg Test (9HPT), Functional Dexterity Test (FDT), and the 6-Minute Walk Test (6MWT).

**Figure 6:**
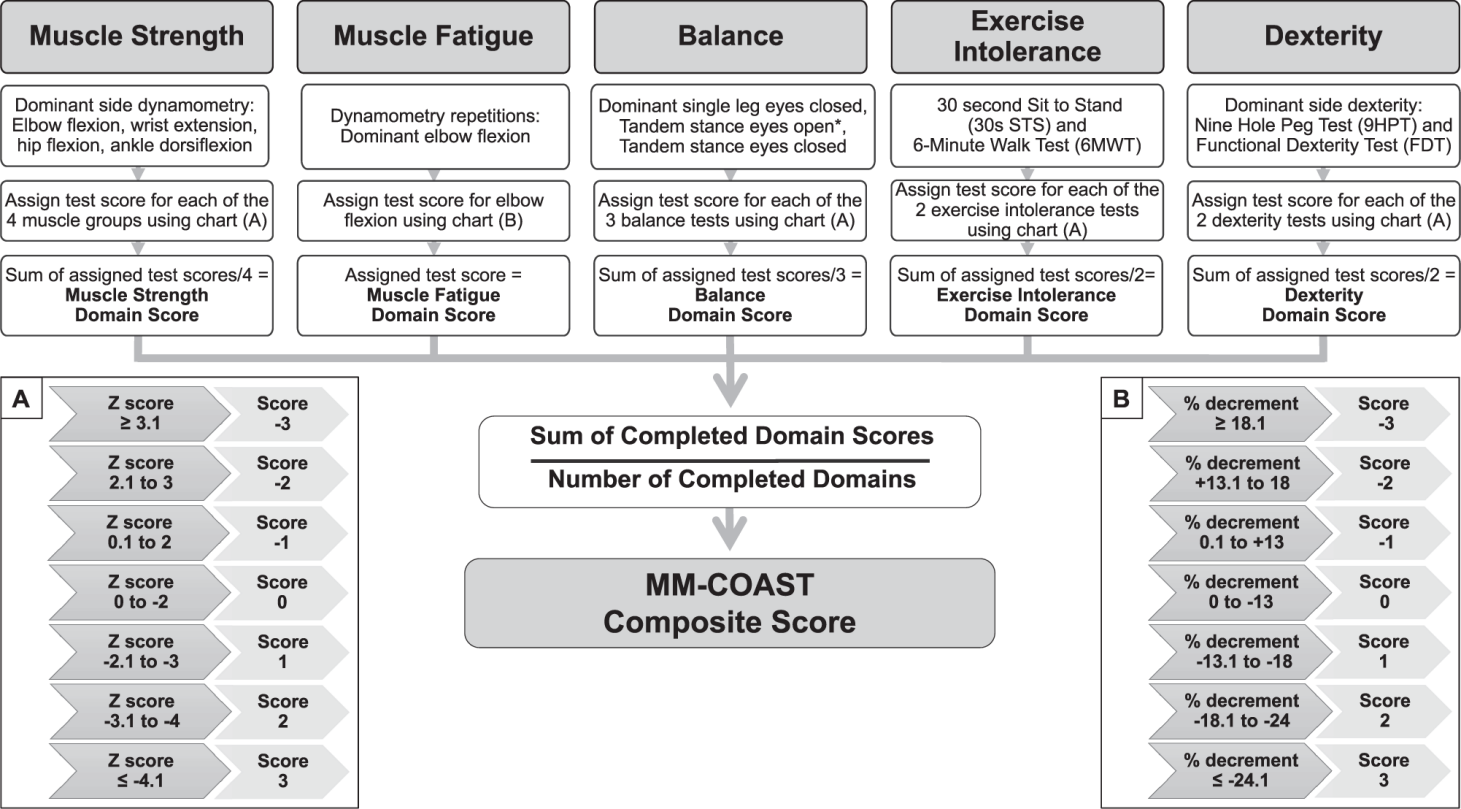
MM-COAST Composite Score. Approach to scoring the MM-COAST for a composite score is shown. Test scores are assigned for each domain assessment raw score, based on *z*-score (chart A) or % decrement for muscle fatigue only (chart B), summed and averaged to achieve a domain score. The mean domain score is presented as the MM-COAST Composite Score. *For participants with a normative SD of 0, use Table S5–A for Tandem Stance Eyes Open assigned score.

The MM-Composite Assesment Tool (MM-COAST) (Figure 5) was developed using objective measures selected from the study objective measures (Figure 1, Table S2). An MM-COAST Composite Score was developed (Figure 6, Table S5). Statistical analyses were conducted in Prism (Version 8.3, San Diego) and RStudio (RStudio Team (2015). RStudio: Integrated Development for R. RStudio, Inc., Boston, MA. http://www.rstudio.com/). Demographics, biochemical results, and objective measures were summarized by standard descriptive statistics, for example, mean ± standard deviation (SD) for continuous variables, and count and percentage for categorical variables. Group comparisons for continuous variables were performed using two-sample *t*-test or ANOVA or their non-parametric equivalent, as appropriate. Principal component analysis (PCA) was performed to summarize the correlation among dynamometry-measured strength between muscle groups tested. 6MWT minute distance slope analysis was performed to obtain the participant-specific slope, interpreted as average distance walked per minute, using linear mixed-effects model which accounts for within-subject correlation due to repeated measures. We used Pearson’s or Spearman’s correlations as appropriate to assess relationships of objective measures to age, height, and weight, as well as to the North Star Ambulatory Assessment (NSAA) functional mobility scale, validated as a clinically meaningful measure in DMD,^22,23^ in order to validate MM objective assessments, as a gold standard functional measure in MM does not exist. Test–retest reliability of the MM-COAST Composite Score was assessed by intraclass correlation coefficient (ICC), and a measure of consistency across MM-COAST domains was assessed by Cronbach’s alpha. Area under the receiver operator curve (AUROC) to determine potential roles of Growth Differentiation Factor 15 (GDF-15), lactate, creatine kinase (CK), and MM CompositeScore as diagnostic biomarkers was calculated using pROC package^24^ in RStudio, as well as the sensitivity and specificity at an established threshold with 95% confidence interval (CI) estimated based on 2,000 bootstrap replicates.

## Results

### Demographics

In the Definite MM group, 35/59 (59%) were children < 18 years of age, 26/59 (44.1%) male (Table 1). Nuclear gene aetiologies were identified in *n* = 18/59 (30.5%), while 41/59 (69.5%) harboured mtDNA aetiologies (Table S1). The most frequent genetic aetiology was single large scale mtDNA deletion (*n* = 13), which were sporadic except in one participant with a co-existing autosomal dominant *de novo* mutation in *SSBP1*.^25^ Diverse inheritance patterns were identified, including X-linked (*AIFM1*).^26^ In the Unlikely group, 14/32 (44%) were children and 12/32 (37.5%) male. Mean body mass index (BMI) in children was 18.0 ± 4.5 in Definite MM and 22.1 ± 6.4 in the Unlikely group, *p* = 0.04. For adults, the mean BMI was 24.6 ± 6.5 in Definite MM and 24.4 ± 9.3 in the Unlikely group, *p* = 0.89 (Table 1).

### Biochemical analyte results

Mean plasma CK was 162.3 ± 16.6 and 90.9 ± 10.0 IU/L (*p* = 0.0003), and venous lactate was 2.0 ± 0.2 and 1.1 ± 0.1 mmol/L (*p* < 0.0001), in the MM and Unlikely groups, respectively (Table 1). Growth Differentiation Factor 15 (GDF-15), previously proposed to be a potential biomarker of mitochondrial disease,^27,28^ was 1,655 ± 262.2 pg/mL (Mayo Clinic Laboratories, normal ≤ 750 pg/mL) in MM (*n* = 44) and 524.9 ± 50.7 pg/mL in the Unlikely group (*n* = 20), *p* = 0.003. GDF-15 levels were highest in participants with Kearns-Sayre Syndrome (KSS, *n* = 12), at 3,093 ± 545.4 pg/mL as compared to other mtDNA and nuclear genetic aetiologies (*p* < 0.001). In four participants with the m.8344A > G mutation (Table S1) with myopathy, GDF-15 levels were also elevated at 2,428 ± 1,252 pg/mL. The potential of GDF-15 as a MM biomarker was evaluated using AUROC, which was previously reported to be 0.82^28^ and 0.99^27^ in mitochondrial disease patients. In our MM cohort, AUC was 0.731 (95% CI: 0.610-0.853) for GDF-15, 0.745 (95% CI: 0.634–0.855) for CK, and 0.769 (95% CI: 0.653–0.885) for lactate. There was no significant difference between the three AUCs (*p* = 0.90, DeLong method). At the established threshold of 750 pg/mL, the specificity of GDF-15 for MM was 90% (95% CI: 76–100%) and sensitivity was 52% (95% CI: 37–67%). This indicates that GDF-15 has poor discriminatory power in MM. Further, there was no association between GDF-15 levels and MM-dominant ankle dorsiflexion muscle strength (*p* = 0.79, *n* = 35) or the 6MWT minute distance slope assessment of exercise intolerance (*p* = 0.77, *n* = 27). These data demonstrate the persistent lack of reliable biochemical diagnostic markers, or meaningful surrogate biomarkers in MM and further highlight the need to validate sensitive and specific myopathy outcome measures that directly measure over short intervals how MM patients feel and function.^29^

## Domain specific mitochondrial myopathy objective measurement

### Dynamometry muscle strength assessments demonstrate that mitochondrial myopathy participants have both proximal and distal muscle weakness

Mitochondrial myopathy proximal and distal muscle weakness was quantified by dynamometry. In the MM cohort, 36/59 (61%) were found to have proximal muscle weakness on clinician neurologic exam. However, dynamometry-revealed muscle weakness (*z*-score < −2 SD) was present in at least one dominant muscle group in 56/59 (94.9%) MM participants, including 20/56 (35.7%) MM participants without weakness on clinician exam. Dynamometry identified a mean sum of 5.1 (range 1–10) dominant muscle groups that were weak (*z*-score <−2 SD) in each MM participant.

In the upper extremities of MM participants (*n* = 59), dominant elbow flexion (mean *z*-score ± SD, −2.6 ± 2.1), wrist extension (−3.4 ± 1.7), gross grasp (−2.1 ± 1.1), and pinch (−2.5 ± 2.8) were weak (Figure 2A, Table S6). In the lower extremities, MM participants (*n* = 59) had weakness of hip flexion (−2.5 ± 2.3), knee flexion (−2.8 ± 1.3), and ankle dorsiflexion (−2.4 ± 2.5) (Figure 2D, Table S6). These results confirm that Definite MM participants in this study had proximal muscle weakness, considered typical of MM.^30^ Unexpectedly, the distal muscles of the upper (wrist extension) and lower (ankle dorsiflexion) extremities were also weak, at *z*-score < −2 SD, demonstrating for the first time that a pattern of both proximal and distal muscle weakness exists in MM (Figures 2A and 2D). Indeed, wrist extension (−3.4 ± 1.7) was the weakest muscle group across the MM cohort comparedto elbow flexion (−2.6 ± 2.1), *p* = 0.018 (Table S6). Distal weakness could not be solely attributed to peripheral neuropathy as only 7/19 (36.8%) MM participants with distal weakness who had a nerve conduction study (NCS) performed due to clinical suspicion, were confirmed to have peripheral neuropathy.

In children with MM (*n* = 35), elbow flexion (−2.3 ± 1.1), wrist extension (−2.6 ± 1.4), gross grasp (−2.2 ± 1.2), and pinch (−2.6 ± 3.0) in the upper extremities, as well as hip (−3.0 ± 1.4) and knee flexion (−2.8 ± 1.3) in the lower extremities, were the weakest muscle groups (Figures 2B and 2E, Table S7). In adult MM (*n* = 24), elbow flexion (−3.2 ± 3.0), wrist extension (−4.6 ± 1.4), and ankle dorsiflexion (−3.8 ± 2.6) were weakest (Figures 2C and 2F, Table S7). Adults with MM had significantly greater distal weakness than child MM, at both wrist extension (−4.6 ± 1.4 vs. −2.6 ± 1.4, *p* < 0.0001) and ankle dorsiflexion (−3.8 ± 2.6 vs. −1.3 ± 1.7, *p* = 0.0004, Table S7) suggesting the possibility of age-related progressive muscle involvement in MM.

There were 6/59 (10.2%) non-ambulatory MM participants, as defined by being unable to take = five steps independently.^11^ Mean muscle strength *z*-scores were significantly lower in participants who were non-ambulant compared with those who were ambulant for dominant side hip flexion (−4.3 ± 1.5, *n* = 4; −2.4 ± 2.3, *n* = 47, *p* = 0.041) and trended lower for elbow flexion (−3.9 ± 2.5, *n* = 6; −2.5 ± 2.1, *n* = 52, *p* = 0.09), wrist extension (−4.6 ± 1.3, *n* = 4; 3.3 ± 1.7, *n* = 48, *p* = 0.14), and ankle dorsiflexion (−4.7 ± 3.3, *n* =4; −2.2 ± 2.4, *n* = 48, *p* = 0.16), respectively (Table S8).

No significant difference in strength was observed between genders or genetic aetiology categories. Specifically, wrist extension (*p* = 0.21, *n* = 55) and ankle dorsiflexion (*p* = 0.80, *n* = 53, ANOVA) muscle strength did not differ between MM participants with nuclear gene disorders, mtDNA pathogenic variants, or mtDNA single large scale deletion syndromes. Correlation analyses revealed (i) that a symmetric pattern of muscle weakness occurs in MM (Figure S1), and (ii) that strength in most muscle groups correlates positively with age, height, and weight, *p* < 0.05, with the exception of three muscle groups that correlated with only height and/or weight (*p* < 0.01, all *r*-values listed in Table S9). PCA revealed that strength in all muscle groups except for neck flexion and pinch were closely correlated (Figure S2). No correlation was observed between the sum of weak muscle groups and assessments of exercise intolerance by either 30s STS (*r* = 0.19, *p* = 0.39, *n* = 23) or 6MWT minute distance slope (*r* = 0.21, *p* = 0.17, *n* = 45), indicative of the relatively low burden of weak muscle groups, at a mean of 5.1 weak (*z*-score < 2 SD) muscle groups per MM participant.

### Static balance assessments revealed significant imbalance occurs in mitochondrial myopathy

The neurologic basis of imbalance in MM includes cerebellar and/or sensory ataxia,^31^ peripheral neuropathy,^32^ vestibular dysfunction,^33^ and/or visual impairment.^34^ Cerebellar ataxia is a prominent clinical symptom in MM patients of all genetic aetiologies including *POLG*^35^ and *ADCK3*,^36^ where tandem stance (TS) is difficult. Maintaining TS with eyes closed imposes further challenges, as this requires the participants to rely more heavily on vestibular and proprioceptive input. In the MM cohort, clinician exam revealed 19/49 (38.7%) participants with broad-based gait ataxia. Objective balance testing, which included standing tandem for up to 10 or 20 seconds depending on age,^20^ identified 37/49 (75.5%) MM participants with imbalance, including 18/37 (48.6%) in whom no gait ataxia was documented on clinician exam.

Significant deficits in TS Eyes Open (−3.2 ± 8.8, *n* = 53, mean *z*-score ± SD) and TS E Closed (−2.6 ± 2.7, *n* = 52) were noted in MM as respectively compared to the Unlikely group (−0.6 ± 6.1, *n* = 32), *p* = 0.02 and (−0.5 ± 1.4, *n* = 32), *p* < 0.0001 (Figure 3A, Table S10). Ability to stand on one leg was assessed as it is essential for activities of daily living (ADLs) such as stair climbing and dressing.^37^ Interestingly, Single Leg Stance Eyes Closed was within the normal range in the overall Definite MM group (Figure 3A, Table S10).

However, significant differences were uncovered when results were analysed by age group. Adults with MM revealed significant difficulty with TS Eyes Open (−5.8 ± 11.6, *n* = 28) (Figure 3C, Table S7), while children with MM performed within the normal range (−0.4 ± 1.0, *n* = 25), *p* = 0.033 (Figure 3B, Table S7). On the other hand, TS Eyes Closed (adults −2.0 ± 1.7, *n* = 28, child −3.2 ± 3.4, *n* = 24, *p* = 0.31) and Single Leg Stance Eyes Closed (adults 1.3 ± 1.0, *n* = 28, child −2.0 ± 1.6, *n* = 25, *p* = 0.10) trended more difficult in child MM (Figures 3B and 3C, Table S7), likely related to greater reliance of this age group on vision compared to vestibular and proprioceptive inputs.^38–40^

Lower extremity muscle strength influences balance, as has been reported in healthy adults,^41^ elderly adults,^42^ and athletes.^43^ In our MM cohort, dominant ankle dorsiflexion strength moderately correlated with TS Eyes Open (Pearson’s correlation coefficient *r* = 0.6, *p* < 0.0001, *n* = 37), while knee flexion strength (*r* = 0.64, *p* = 0.002, *n* = 21) moderately correlated with TS EyesClosed. Fatigue has also been shown to influence balance in athletes.^44^ In our MM cohort, TS Eyes Closed (*r* = 0.44, *p* = 0.03, *n* = 25) moderately correlated with 30s STS. In addition, TS Eyes Open (*r* = 0.37, *p* = 0.017, *n* = 40) and TS Eyes Closed (*r* = 0.33, *p* = 0.037, *n* = 39) weakly correlated with 6MWT minute distance slope as well as 6MWT *z*-scores [TS Eyes Open (*r* = 0.34, *p* = 0.034, *n* = 40) and TS Eyes Closed (*r* = 0.47, *p* = 0.002, *n* = 39, Table S11)]. Overall, these data indicate that ankle weakness, knee flexion weakness, and exercise intolerance are related to MM imbalance.

### Dexterity assessments showed significant deficits in mitochondrial myopathy

Both FDT [−5.9 ± 6.0, *n* = 44 (mean *z*-score ± SD)] and 9HPT (−8.3 ± 11.2, *n* = 53) performance was significantly below the expected mean across all ages in MM. In the Unlikely group, FDT (−3.1 ± 3.4, *n* = 25) and 9HPT (−2.9 ± 3.9, *n* = 26) were also below expected mean but significantly less impaired compared to MM participants (*p* = 0.033 and *p* = 0.006 respectively, Table S10, Figure 3D).

In our MM cohort, 5 of 16 participants (31.2%) with normal 9HPT scores, had abnormal FDT scores (*z*-score < 2 SD). This is consistent with prior studies that suggest the FDT is able to identify more subtle deficits than simpler grasp and release dexterity tests.^45^ No significant difference was seen in FDT or 9HPT performance between adult MM (*n* = 22, FDT; *n* = 24, 9HPT, Figure 3F) and child MM (*n* = 21, FDT; *n* = 29, 9HPT, Figure 3E), *p* = 0.18 and *p* = 0.47, respectively (Table S7).

Correlation of grip strength with dexterity has previously been reported in healthy adults^46^ and children^47^ measured with the 9HPT, but not when evaluated by FDT.^48^ In the MM group, dominant 9HPT weakly correlated with dominant gross grasp (*r* = 0.33, *p* = 0.04, *n* = 40), but did not correlate with dominant pinch strength (*r* = 0.22, *p* = 0.30, *n* = 25). Indeed, FDT did not correlate with either dominant gross grasp (*r* = 0.10, *p* = 0.57, *n* = 32) or pinch strength (*r* = 0.27, *p* = 0.28, *n* = 18). This suggests that MM dexterity assessments do not rely on muscle strength. When compared to balance assessments, 9HPT positively correlated with TS Eyes Closed (*r* = 0.32, *p* = 0.034, *n* = 43), while FDT correlated with both TS Eyes Open (*r* = 0.45, *p* = 0.004, *n* = 39) and TS Eyes Closed (*r* = 0.33, *p* = 0.041, *n* = 39). In addition, 9HPT weakly correlated with 6MWT slope (*r* = 0.32, *p* = 0.043, *n* = 40) and moderately correlated with 6MWT *z*-scores (*r* = 0.40, *p* = 0.011, *n* = 40). Similarly, FDT weakly correlated with 6MWT slope (*r* = 0.33, *p* = 0.044, *n* = 38) and 6MWT *z*-scores (*r* = 0.36, *p* = 0.026, *n* = 38). In summary, these results indicate that exercise intolerance as well as imbalance is associated with impaired dexterity, and both FDT and 9HPT should be assessed to fully capture the range of deficits in MM dexterity. The 9HPT is an easier test and allows capture of data from participants unable to complete the FDT due to significantly impaired in-hand manipulation skills and/or poor cognition, thus avoiding a high floor effect. Conversely, only assessing the 9HPT would result in a ceiling effect, as participants with less impaired dexterity would easily complete the 9HPT.

### Exercise intolerance is a quantifiable outcome in mitochondrial myopathy

All MM participants self-reported exercise intolerance at clinic visits. The 30 second Sit to Stand (30s STS)^20^ and 6-minute walk test (6MWT)^20^ assessments were used to objectively measure exercise intolerance, with 30s STS being a strength-based measure^49,50^ and the 6MWT a mobility-based assessment. Mean *z*-score for 30s STS in MM was borderline abnormal at −2.0 ± 0.8, mean ± SD, *n* = 38, and not significantly different to −1.7 ± 0.9, *n* = 17, in the Unlikely group, *p* = 0.14 (Table S10). When analysed by age group, adult MM mean *z*-score was abnormal at −2.1 ± 0.7, *n* = 16, while child MM mean *z*-score was borderline at −2.0 ± 0.8, *n* =22 (Figure 4A, Table S7). In the MM cohort, 30s STS was not associated, by Pearson correlation, with dominant muscle strength for hip flexion (*r* = 0.03, *p* = 0.91, *n* = 22) or hip abduction (*r* = 0.08, *p* = 0.70, *n* = 25, Table S11). 30s STS primarily relies on knee extensor muscles. As knee extension strength was only measured in the child MM group, no correlation was identified in the small number of participants (*r* = 0.02, *p* = 0.94, *n* = 14, Table S11). A moderate correlation was found between 30s STS and balance testing, TS Eyes Closed (*r* = 0.44, *p* = 0.03, *n* = 25, Table S11).

Six-minute walk test was performed to measure both the total distance walked in six minutes^20^ and the distance walked each minute.^21,51^ The total distance walked, measured in metres (m), was significantly shorter in the MM cohort (425.3 ± 13.7 m, mean ± SEM, *n* = 46) compared with the Unlikely group (494.4 ± 24.4 m, *n* = 28), *p* = 0.011 (Table S10). The mean percent predicted total distance walked by McKay *et al*. predictive equation ^20^ was 63.6 ± 2.1% (mean % predicted ± SEM) for the MM cohort and 74.5 ± 3.7% for the Unlikely group, *p* = 0.012 (Table S10). However, no correlation was found between total distance walked and age, height, weight, or gender in the MM participants (Figure S3). This is surprising, as numerous predictive equations include a combination of height, weight, and age as was utilized here, for example for females ages 3–19 years: −54.9+ [1.1*age (years)] + [5.5*height (cm)] – [2.7*weight (kg)].^20^ To emphasize this further, we applied diverse prediction equations from the literature to our MM 6MWT data and observed no significant differences in the mean % predicted values regardless of whether the equation incorporated any combination of age, height, weight, or gender using McKay (age, height, and weight),^20^ Halliday (age, height, and weight),^52^ Enright (age, height, and weight),^53^ and Ulrich (height-based, weight-based, and age-based)^54^ equations, (*p* = 0.22, Kruskal–Wallis, *n* = 6−16, data not shown). Thus, we recommend avoiding the use of published 6MWT prediction equations from the literature in MM.

No correlation was identified between total distance walked and dominant ankle dorsiflexion strength (*r* = 0.31, *p* = 0.071, *n* = 35) or hip flexion strength (*r* = 0.31, *p* = 0.08, *n* = 34, Table S11). In addition, there was no correlation to the 30s STS (*r* = 0.33, *p* = 0.13, *n* = 23), as previously reported in healthy adults.^55^ Overall, these results indicate that the 6MWT expressed as total distance walked, although decreased in MM, does not provide a meaningful measure of MM exercise intolerance.

However, when 6MWT was analysed as *z*-scores using published normative data,^20^ the Definite MM group was abnormal (−2.9 ± 1.3, mean ± SD, *n* = 46) and significantly different compared to the Unlikely group (−1.8 ± 1.6, *n* = 28), *p* = 0.001, and there was a strong correlation to 30s STS (*r* = 0.80, *p* < 0.0001, *n* = 30), moderate correlations with dominant ankle dorsiflexion strength (*r* = 0.67, *p* < 0.0001, *n* = 39), balance testing TS Eyes Closed (*r* = 0.47, *p* = 0.002, *n* = 39), and 9HPT (*r* = 0.40, *p* = 0.011, *n* = 40), and weak correlations with FDT (*r* = 0.36, *p* = 0.026, *n* = 38) and dominant elbow flexion strength (*r* = 0.36, *p* = 0.03, *n* = 37, Table S11), indicating clinical meaning of the MM 6MWT data when expressed as *z*-scores.

Alternative analysis of minute-to-minute 6MWT distances by linear mixed-effects model uncovered a decline in distance walked over time in both cohorts, with only the MM group reaching statistical significance (slope = −0.9, *p* = 0.03, *n* = 46) but not the Unlikely group (slope = −1.39, *p* = 0.70, *n* = 28, Figure 4E). A moderate correlation was observed between 6MWT slope and dominant ankle dorsiflexion strength (*r* = 0.41, *p* = 0.013, *n* = 35) suggesting that increased distal muscle weakness was associated with a progressive decline in minute distances. Correlations were also found with dexterity (9HPT, *r* = 0.32, *p* = 0.043, *n* = 40; FDT, *r* = 0.33, *p* = 0.044, *n* = 38) and balance (TS Eyes Open, *r* = 0.37, *p* = 0.017, *n* = 40; TS Eyes Closed, *r* = 0.33, *p* = 0.037, *n* = 39). These results indicate that 6MWT minute distance slope analysis also provides a meaningful measure of exercise intolerance. The 6MWT slope analysis did not correlate to 30s STS (*r* = 0.34, *p* = 0.12, *n* = 23), which suggests that 30s STS and 6MWT minute distance assessments provides distinct measures of exercise intolerance.

In summary, the 6MWT data is meaningful when presented as *z*-scores using published normative data, or by minute distance slope analysis, while 6MWT predictive equations should be avoided in MM.

### Muscle fatigue is quantified by dynamometry repetitions assessment

Patients with MM frequently report muscle strength that decreases with continuous or consecutive muscle contractions. The ability to quantify muscle fatigue would be of utmost importance in future longitudinal studies and clinical trials. Novel analyses of the decrement in muscle strength (in pounds) on the sixth as compared with the first dynamometry repetition [mean negative percent decrement expressed as percentage (%) ± SEM] in MM revealed a decrement of −14.7 ± 2.2% (*n* = 21) in the dominant elbow flexors and −15.4 ± 3.7% (*n* = 21) in the non-dominant elbow flexors. A mean decrement of −10.9 ± 4.4% (*n* = 19) was observed in the dominant hip flexors and −11.6 ± 4.8% (*n* = 18) in the non-dominant hip flexors (Figure 4B, Table S10). When results were analysed by age group, mean negative percent decrement at dominant elbow and hip flexion were similar in adult compared to child MM participants, *p* = 0.57 and *p* = 0.62, respectively (Table S7). In a clinical trial, results of this negative percent decrement in dynamometry repetitions assessment could be compared before and after the target intervention in each individual, eliminating the need for normative population data. Negative percent decrement in MM participants did not correlate with their dominant hip flexion (*r* = −0.28, *p* = 0.25, *n* = 19), or dominant elbow flexion (*r* = −0.13, *p* = 0.59, *n* = 21) strength (Table S11). This is consistent with our clinical observation that MM participants report varying degrees of fatigable weakness on serial repetitions, despite normal baseline muscle strength. Furthermore, no correlation in MM mean negative percent decrement was observed with 30s STS or 6MWT (*z*-scores or slope analysis), suggesting dynamometry repetition analysis offers a discrete assessment of fatigable weakness in MM.

As anticipated, we observed a mean negative percent decrement of −20.0 ± 5.4% at dominant elbow flexion in the Unlikely group (*n* = 8,Table S10), as these participants also reported muscle fatigue. We attempted to characterize the normal muscle fatigue response for this measure by conducting assessments in an exploratory healthy volunteer cohort. In healthy adult and child volunteers [*n* = 25, mean age 26.2 ± 14.3 years, 9/25 (36%) male], mean negative percent decrement at dominant elbow flexion was −6.5 ± 2.8% (*n* = 24), which was significantly different to MM participants (−14.7 ± 2.2%, *n* = 21), *p* = 0.036. Mean negative percent decrement at dominant hip flexion was 1−0.0 ± 1.6% (*n* = 24), similar to MM participants (−10.9 ± 4.4%, *n* = 19), *p* = 0.47.

Analysis of mean negative percent decrement of this exploratory healthy volunteer cohort by age groups is presented in Tables S7 and S10. A notable finding was that the healthy child (<18 years) volunteers, (*n* = 10, mean age 11.2 ± 3.5 years, 50% female) mean negative % decrement for dominant elbow flexion (−6.5 ± 1.9%, *n* =9) was significantly different to the child MM participants (−15.39 ± 3.2%, *n* = 12, *p* = 0.034).

It is important to emphasize that the magnitude of negative percent decrement (mean *z*-score ± SD) in MM participants should be interpreted in the context of the muscle strength *z*-scores at the first and sixth repetitions, which truly demonstrate the phenomenon of fatigable muscle weakness of MM. Here, the raw muscle strength data obtained at the first and sixth repetitions are compared to normative data obtained in the non-fatigued state^16,17^ to show decreased muscle force in fatigued muscle on the sixth repetition, and presented as *z*-scores (Figure 4C). In the MM cohort, mean *z*-score was within normal limits on the first repetition for dominant elbow flexion (−1.8 ± 1.8, *n* = 22), and declined significantly in strength on the sixth repetition (−2.6 ± 1.7, *n* = 21, *p* < 0.0001), (Table S10, Figure 4C). There was also significant decline of the muscle strength *z*-scores at dominant hip flexors between the first (−1.8 ± 2.0, *n* = 25) and sixth (−2.2 ± 1.8, *n* = 19) dynamometry repetitions, *p* = 0.021 (Figure 4C, Table S10). Similar results were observed at non-dominant elbow and hip flexion (Table S10). Thus, in the MM group, the mean muscle strength *z*-score was within normal limits (*z*-score ≥ −2 SD) on the first repetition and declined to an abnormal *z*-score < −2 SD on the sixth repetition for dominant elbow and hip flexion, which indicates fatigable muscle weakness that is typical of MM.

In contrast, in the healthy adult and child volunteers, mean *z*-score was within normal limits on the first repetition for dominant elbow flexion (0.3 ± 1.5, *n* = 24) and dominant hip flexion (0.4 ± 1.7, *n* = 24) and remained within normal limits on the sixth repetition for dominant elbow (−0.4 ± 1.6) and hip (−0.5 ± 1.6) flexion (Table S10). Results were similar on the non-dominant sides and when analysed by adult and child age groups (Table S10).

We next evaluated the potential utility of dynamometry repetitions assessments to discriminate MM from healthy individuals. AUROC analysis revealed an ideal threshold (modified Youden index) of −13% at both elbow flexion with a specificity of 75% and sensitivity of 65%, and at hip flexion with a specificity of 75% but lower sensitivity of 60%. Hence, we designated a cut-off of < −13% decrement to be abnormal at elbow flexion in MM, as indicated in the MM-COAST composite score (Figure 6). Dynamometry repetitions assessment presented as percent decrement should not be utilized exclusively as a diagnostic tool to define MM.

### Objective assessments for mitochondrial myopathy-composite assessment tool and development of a mitochondrial myopathy composite score

The mean assessment time to complete all MM objective assessments in this study was ~75–90 minutes. Based on the results of this study, we constructed the MM-COAST (Figure 5), intended for objective assessment of MM participants in a longitudinal study or intervention clinical trial. It is important to highlight that the MM-COAST was not developed as a diagnostic tool for MM. The MM-COAST incorporates these objective assessments across five domains, conducted in descending order: dynamometry assessment of muscle strength, dynamometry repetitions of elbow flexion, static balance tests (Single Leg balance Eyes Closed and TS Eyes Open and Closed), 30s STS, 9HPT, FDT, and the 6MWT, all expressed as *z*-scores except dynamometry repetitions, expressed as negative percent decrement (Figure 5). Only elbow flexion was included for dynamometry repetitions assessment as it was more reliably performed across all ages compared with hip flexion repetitions. In addition, the 6MWT can be expressed as minute distance slope analysis, where normative data is not required, and cohort-level data can be compared following intervention in a clinical trial.

We developed as an option, the MM-COAST Composite Score, outlined in Figure 6 and Table S5. In this study, 53/59 (89.8%) MM participants were able to attain a composite score, including 4/6 (66.7%) who were non-ambulatory. Here, MM-COAST dynamometry scores are limited to dominant side elbow flexion, wrist extension, hip flexion, and ankle dorsiflexion to represent overall MM muscle strength, as muscle weakness in MM is symmetrical (Figure S1) and PCA confirmed that all muscle groups tested were correlated except neck flexion and pinch (Figure S2). These four muscle groups were selected because they were identified as the weakest muscles in the upper and lower extremities in our MM cohort. Similarly, only dominant side assessments of Single Leg balance Eyes Closed and dexterity assessments (9HPT and FDT) were included in the composite score. Thus, time to complete the MM-COAST Composite Score assessment will be reduced to ~60 minutes.

The mean (±SEM) MM Composite Score for the MM group was 1.3 ± 0.1 (*n* = 53), which was significantly different when compared to the Unlikely group composite score 0.5 ± 0.2 (*n* = 29), *p* = 0.0005, with a higher MM Composite Score indicating greater MM disease severity (Figure 4F, Table S10). Indeed, the mean composite score for the non-ambulatory MM participants (2.7 ± 0.17, *n* = 4) was significantly higher than the ambulatory MM participants (1.2 ± 0.8, *n* = 49, *p* = 0.0001, Table S8). In the ambulatory participants, 44/53 (83.0%) fully completed assessments in ≥ 4 domains. Of those who completed assessments in five domains, 15/19 (78.9%) had abnormal z-scores in ≥ 3 domains. Likewise, of those who completed four domains, 19/25 (79.2%) had abnormal *z*-scores in ≥ 3 domains. In the ambulatory participants who only completed three domains, 4/5 (80%) had abnormal *z*-scores in all three domains. Thus, it is recommended that where possible, objective assessments in all domains are completed to ensure that the MM-COAST Composite Score is reflective of diverse MM domains, with a minimum completion in three MM-COAST domains in ambulatory individuals, and at least two domains in non-ambulatory individuals where muscle strength, muscle fatigue, and dexterity assessments are most likely to be achieved.

Mitochondrial Myopathy Composite Scores correlated with all MM-COAST domains, except for muscle fatigue repetitions assessment likely related to the relatively small cohort size tested, and to the NSAA (*r* = −0.64, *p* < 0.0001, *n* = 52, Table S11). MM-COAST Composite Scores did not correlate with plasma CK (*r* = −0.07, *p* = 0.67, *n* = 40), lactate (*r* = 0.16, *p* = 0.73, *n* = 38) or GDF-15 (*r* = 0.16, *p* = 0.39, *n* = 30) levels. In those with mtDNA-disease, there was no correlation to mtDNA heteroplasmy levels in muscle (*r* = −1.00, *p* = 0.33, *n* = 3), blood (*r* = 0.12, *p* = 0.62, *n* = 19) or buccal tissue (*r* = −0.24, *p* = 0.58, *n* = 8). Test-retest reliability assessment of the MM-COAST Composite Score performed in a subset of MM participants (*n* = 14) revealed a good intraclass correlation coefficient (ICC) of 0.81 (95% CI: 0.59–0.92). An assessment of internal consistency revealed an acceptable Cronbach’s alpha of 0.67 across all five domains (*n* = 22), which was anticipated as the individual MM domains are distinct.

We assessed the utility of MM-COAST as a potential diagnostic tool in defining MM. This revealed an AUC of 0.743, while earlier analyses of biochemical analytes revealed AUC of 0.732 for GDF-15, 0.745 for CK and 0.805 for lactate. The ideal threshold for the MM composite score was 0.41 (modified Youden index), with a specificity of 61% and sensitivity of 84%. These results indicate that the MM-COAST Composite Score is less specific than GDF-15 levels but is more sensitive as a diagnostic tool than plasma CK and GDF-15. Hence, the MM-COAST and composite score can be utilized as an objective tool to measure disease severity over time and following intervention in a clinical trial, but not as an independent diagnostic tool. Given its high sensitivity, the MM-COAST Composite Score may carry potential as a diagnostic screening tool, if incorporated in a future multimodal diagnostic application, in order to differentiate ’Definite’ compared to ’Unlikely’ MM individuals.

## Evaluation of the clinical meaningfulness of dynamometry, dexterity, fatigue, and balance assessments

### Comparison of MM objective assessments to the North Star Ambulatory Assessment (NSAA) of motor function

The NSAA is a strength-based functional mobility scale developed for administration in ambulant boys with Duchenne Muscular Dystrophy (DMD),^22^ as young as 3 years.^22,56^ Typically developing children with independent motor function should attain an NSAA full score of 34,^57^ while an NSAA score of 13 predicts DMD loss of ambulation within 24 months from the time of measurement.^58^ In our MM cohort, NSAA revealed a mean score of 25.1 ± 1.3, mean ± SEM (*n* = 58, Table S10), which would be equivalent to the mean score of a 7-to 8-year-old boy with DMD on daily prednisolone.^59^ No difference was observed in adult MM as compared to child MM mean NSAA scores (Table S7). These results indicate that MM individuals maintain a higher level of independent motor function compared to individuals with DMD, consistent with the slower and less predictable disease course in MM. Nonetheless, when compared with the significantly higher Unlikely MM group mean score of 29.8 ± 1.1, (*P* = 0.006, *n* = 31), the NSAA score in our MM cohort indeed reflects diminished motor function (Figure 4D, Table S10).

The most challenging NSAA items for the MM cohort which participants were unable to execute or required some compensation (scored 0 or 1 out of 2 possible points) included standing on right leg for 3 seconds (36/58, 62%), rising from the floor (24/58, 41.4%), running for 10 metres (36/52, 69.2%), and hopping once on right leg (23/57, 40.4%, Table S10). This is consistent with hip girdle weakness and imbalance. Indeed, a positive correlation was observed between NSAA scores and dominant hip flexion (*r* = 0.33, *p* = 0.028, *n* = 46) and dominant hip abduction muscle strength (*r* = 0.37, *p* = 0.009, *n* = 48), as well as with TS Eyes Open balance assessment, (*r* = 0.54, *p* < 0.0001, *n* = 44). Standing on heels for 3 seconds was also difficult in the MM cohort (27/57, 47.4%), and there was a moderate correlation between NSAA scores and dominant ankle dorsiflexion strength (*r* = 0.54, *p* < 0.0001, *n* = 45). NSAA scores also moderately correlated with dominant wrist extension strength (*r* = 0.41, *p* = 0.005, *n* = 45), 30 s STS (*r* = 0.48, *p* = 0.012, *n* = 27), 6MWT total distance walked (*r* = 0.67, *p* < 0.0001, *n* = 40), 6MWT minute distance slope (*r* = 0.68, *p* < 0.0001, *n* = 40), and 6MWT *z*-scores (*r* = 0.55, *p* < 0.0001, *n* = 44) (Table S11). Overall, these results demonstrate that MM motor function is negatively impacted by weak muscle groups (specifically hip flexion, ankle dorsiflexion, and wrist extension that were shown to be weak in this MM cohort) as quantified by dynamometry; as well as imbalance and exercise intolerance, as quantified by balance assessments, 30 s STS, and 6MWT, respectively, of the MM-COAST; and thus confirms the clinical meaning of these MM objective assessments. Although hand dexterity is not directly assessed in the NSAA, a moderate correlation was observed between NSAA scores and both 9HPT (*r* = 0.56, *p* < 0.0001, *n* = 46) and FDT (*r* = 0.50, *p* = 0.001, *n* = 39). Most importantly, a moderate correlation was observed with MM-COAST Composite Scores (*r* = −0.64, *p* < 0.0001, *n* = 52), providing evidence of the clinical meaning of the MM-COAST.

## Discussion

Here, we have validated an MM-Composite Assessment Tool (MM-COAST) that fully captures the mutually interactive MM key domains of muscle strength, muscle fatigue, balance, dexterity, and exercise intolerance in individuals with MM to measure disease severity over time and following intervention in a clinical trial. In contrast to the NMDAS which lacks objective measures and reflects multisystem disease, the MM-COAST focuses on myopathy and consists of (i) dynamometry assessment of dominant side elbow flexion, wrist extension, hip flexion, and ankle dorsiflexion, (ii) dynamometry repetitions of dominant elbow flexion, (iii) static balance tests (Single-Leg Stance Eyes Closed and TS Eyes Open and Closed), (iv) the 30s STS for exercise intolerance, the (v) 9HPT and FDT for dexterity, and the (vi) 6MWT performed in that order (Figure 5). Reproducibility was demonstrated by the test–retest reliability score of the MM-COAST Composite Scores. The carefully scripted order of assessments was well tolerated by MM participants. A summary of the correlations and clinical meaning, as well as the rationale for inclusion of specific objective assessments in the MM-COAST is summarized in Table S12. Our approach to an MM-COAST Composite Score, representative of the full MM phenotype, was developed (Figure 6). MM-COAST provides a meaningful, quantitative assessment that can be utilized for MM longitudinal studies and future intervention trials. In contrast to other congenital myopathies, MM has greater variation in age of onset, phenotype manifestations and severity, and disease trajectory over time, even within affected kindreds.^60^ Conducting clinical trials in an MM cohort with a single genetic aetiology would not adequately capture the implications for the broader array of genotypes, particularly in mtDNA disorders, which are complicated by tissue heteroplasmy levels.^4^ Indeed, the MM-COAST could facilitate future MM study cohort selection by phenotype, such as patients with a target magnitude of fatigue, weakness, and/or imbalance. The MM-COAST is not intended as an independent diagnostic tool for MM.

This study is the first to quantify muscle strength in MM by hand-held dynamometry, leading to several important findings. First, dynamometry provides more precise, objective, and quantitative information than conventional manual muscle testing by Medical Research Council (MRC) grading.^61^ In our experience, MRC grading does not identify subtle muscle weakness or incremental changes. Indeed, dynamometry identified muscle weakness in *n* = 20/56 (35.7%) of MM participants who displayed no weakness on MRC grading. Second, we demonstrated that symmetric patterns of both proximal and distal muscle weakness occur in MM (Figure S1). These results challenge the dogma that MM causes only, or predominantly, a proximal myopathy.^30^ Indeed, in MM upper extremities, dominant wrist extension (*z*-score −3.4 ± 1.7) was significantly weaker compared to more proximal elbow flexion strength (−2.6 ± 2.1, *p* = 0.018) (Table S6). Third, the weakest muscle groups in MM were 2–3 SD below the expected mean. By contrast, ambulant patients with DMD (5–8 years, *n* = 16) have more severe weakness up to 6.5 SD below expected mean,^62^ and patients with spinal muscular atrophy (5–60 years, *n* = 120) have knee extensor muscle strength that is 5% of predicted reference values, with grip, elbow flexion, and knee flexion strength that is 20% of predicted reference values.^63^ Profound muscle weakness is therefore not a characteristic of MM, nor is MM muscle involvement diffuse, given the mean of only five dominant muscle groups (<50% of all muscle groups tested) were affected in each MM participant.

Lastly, MM adults were significantly weaker at wrist extension and ankle dorsiflexion than were MM children (Table S7), which may be explained by a distinct predilection for progressive skeletal muscle involvement in adult MM. Conventionally, muscle architecture is one of the predictors of muscle function.^64^ While structurally abnormal muscle with resultant muscle weakness is typical of many neuromuscular disorders such as DMD,^56^ muscle architecture in MM is highly variable. The spectrum ranges from normal pathology, to cytochrome oxidase negative fibres,^65^ to ragged red fibres that have mitochondrial proliferation, to variable type I^66,67^ or type II fibre atrophy.^68^ MM symptoms therefore do not predominantly originate from an abnormal muscle structural pathology. Rather, muscle OXPHOS deficiency is the underlying basis of MM, which gives rise to exercise intolerance and fatigue as the more predominant clinical features.

Balance is a complex process, requiring processing of sensory input from the vestibular, proprioceptive, and visual systems. Self-reported imbalance was among the top 5 of 35 multisystem symptoms of mitochondrial disease reported by ~78.8% of patients we previously surveyed.^9^ Here, quantitative assessments identified a similar prevalence, with 37/49 (75.5%) MM participants demonstrating objective evidence of imbalance. Of course, static balance assessments may not fully capture all of the diverse aetiologies contributing to imbalance in MM, including vestibular dysfunction that is discerned by neuro-otologic testing.^33^ Our results also revealed that child MM participants revealed more difficulty with balance assessments with eyes closed than did adult MM participants, with correlation to knee flexor weakness that suggest weaker proximal muscles and reliance on visual compensation contribute to childhood MM imbalance.

Six-minute walk test assessments revealed several pertinent findings in MM. First, although the measured total distance walked in MM (425.3 ± 13.7 m) was significantly shorter compared to the Unlikely group (494.4 ± 24.4 m), *p* = 0.011, the clinical significance of this is not clear.^7^ The total distance walked in the MM group is relatively well preserved when considering listed lung transplant candidates walk only ~335 m in their 6MWT.^69^ Furthermore, the operative risk of a single lobectomy for lung cancer is lower when 6MWT is ≥400 m.^70^ A correlation between 6MWT total distance walked to NSAA total score was identified; however, no correlation between total distance walked and the other objective measures was seen (Table S11). In comparison, the 6MWT *z*-score and minute distance slope analysis both correlate with ankle dorsiflexion strength, balance, and dexterity assessments, as well as NSAA score (Table S12). Therefore, 6MWT would be better expressed as *z*-scores (relative to normative data) or minute distance slope analysis (does not require normative data), which provides a meaningful measure of exercise intolerance in MM.

We also developed and validated in this study a new outcome assessment that utilized six dynamometry repetitions to quantify muscle strength decrement, capturing the fatigable weakness that typifies MM.^35^ Mean MM dominant elbow flexion negative percent decrement was ~2.1–2.3 times higher than in healthy child and adult volunteers, respectively. Although we tested at two muscle groups, elbow and hip flexion, we found that elbow flexion repetitions were more reliably performed compared to hip flexion repetitions across the ages. Therefore, only dominant elbow flexion dynamometry repetitions were included in the MM-COAST. When analysed by first to sixth repetition muscle strength *z*-scores, sixth repetition *z*-scores were in the abnormal range (*z*-score < −2) at the elbows in MM participants (Figure 4C), indicating fatigable muscle weakness, yet remained within the normal range in the healthy adult and child volunteers (*z*-score ≥ −2 SD), where fatigable weakness was not observed (Table S10). Hence, dynamometry repetitions assessment is expressed as percent decrement (incorporated in the MM-COAST Composite Score) and interpreted in the context of the first to sixth repetition muscle strength *z*-scores. We recognize that the number of participants in our study cohort who completed the dynamometry repetitions assessments was relatively low compared to assessments in the other domains. We have subsequently completed this dynamometry repetitions assessment in a second adult and child MM cohort (*n* = 14, mean age 26.5 ± 17.2 years, mean ± SD, 78.6% female). Mean dominant elbow flexion negative percent decrement was found to be −17.04 ± 3.64%. This is comparable to the mean of −14.7 ± 2.2% measured in the Definite MM cohort (*n* = 21) included in this manuscript, and demonstrates the reproducibility of our dynamometry repetitions muscle fatigue assessment.

Both the 30s STS and dynamometry repetition tests involve short bursts of high intensity exercise, which utilize the phosphocreatine shuttle^71^ and anaerobic glycolysis as energy sources. Slow recovery rate of muscle free creatine post-exercise has been reported in PMD, consistent with decreased creatine kinase activity that relies directly on mitochondrial OXPHOS capacity.^72^ Further, oxidative metabolism may contribute significantly during intense exercise bouts of 30–60 second duration.^73^

The NSAA tool that was developed to assess the functional impact of weakness in DMD was utilized in this study as a gold standard assessment tool of motor function against which to compare our MM-COAST assessments. Indeed, NSAA results confirmed the association between function and the physical limitations we identified on MM-COAST testing. These data are also the first to validate NSAA performance in MM, suggesting NSAA offers a meaningful assessment of motor function in MM. However, there was an observed ceiling effect as MM results in a less severe neuromuscular phenotype than DMD. In addition, the NSAA scoring system for strength-and balance- based assessments is not adequately sensitive to capture incremental change in MM therapeutic trials. By contrast, the MM-COAST analysis allows for delineation and quantitation of several MM key domains not assessed by the NSAA.

This study has several limitations. First, normative values for 17- to 19-year-old participants in dynamometry protocols utilized for this study is lacking.^16,17^ As our cohort included three participants in this age group, we utilized 16- year -old normative values (and corresponding testing positions) for 17- year- old participants, and 20- year- old normative values for 18- and 19- year- old participants. In addition, knee flexion and extension in adult MM could not be assessed due to lack of normative population data in the specific protocol used.^17^ Second, as objective assessments such as muscle strength correlate with height and weight, a future study to establish normative data of all MM-COAST assessments in healthy individuals with matching BMI should be conducted. Third, balance assessments indicated a ceiling effect in some MM participants, which is also found in the general population as reflected by a SD of 0 in the normative data of specific age groups.^20^ This may be resolved by extending assessments to 20–30 seconds. A ceiling effect was also observed in the 30s STS test, and a 60s STS test has been considered.^55^ However, normative data through the age-span for these modified assessments would ultimately need to be established. Fourth, the number of participants able to complete each individual domain of the MM-COAST was not uniform throughout all assessments. This is related to MM disease variability, where some participants have more difficulty in specific domains. In anticipation of this, we established a minimum number of assessments that needs to be completed by each participant in order to achieve an MM-COAST Composite Score (Table S5). Fifth, there should be a standardized approach to assessment administration, including ensuring assessments are performed at the same time of day and restricting meals and physical activity immediately prior to assessments. We recommend that MM-COAST assessments are conducted by experienced evaluators, with reliability training being essential prior to its implementation, particularly in multicentre clinical trials. Sixth, the MM composite score provides the benefit of a single, interpretable overview of MM. However, a recognized limitation of composite scores is the anticipation that the final score will not reflect the variation(s) within individual domains. Nevertheless, our data indicate that the majority of MM participants demonstrated abnormal *z*-scores across most domains. Lastly, intra-rater and inter-rater reliability testing was not performed in this study because most assessments were conducted by one physical therapist and only baseline assessments at clinic visits were included in this study. Ongoing reliability assessments are underway.

In summary, we have designed and validated the MM-COAST composite outcome measure to quantify the core domains of MM. We have demonstrated that this composite measure is feasible, tolerable, and clinically meaningful in both adults and children ≥6 years with MM. Using this instrument, we demonstrated for the first time that MM presents with both proximal and distal muscle weakness, significantly impaired hand dexterity, and distinct manifestations of imbalance in child vs. adult MM cohorts. Further, this work demonstrated that 6MWT minute distance slope analysis and dynamometry repetitions are useful new measures to respectively quantify exercise intolerance and muscle fatigue in MM. These data also provide unique insights into the reciprocity and interdependence of core MM domains of weakness, muscle fatigue, imbalance, exercise intolerance, and dexterity; while these domains are interrelated, each may have variable prominence in different MM participants. Having quantitative measures to discriminate the relative contributions of core domains in MM, their response to therapeutic interventions, as well as their objective relation to MM patient function and feeling, will be important in facilitating robust clinical trials for MM. Overall, MM-COAST may hold particular utility as a clinically meaningful composite outcome measure in future MM longitudinal studies and intervention trials.

## Supplementary Material

Supplementary Tables **Table S1.** Mitochondrial Myopathy Genetic Etiologies
**Table S2.** Sequence of Study Objective Measures and References
**Table S3.** Assessment Methods of Study Objective Measures
**Table S4.** Clinical Observations, Advantage and Challenges of Study Assessments.
**Table S5.** MM-COAST Composite Score (See Figure 6)
**Table S5-A.** Test scores for Tandem Eyes Open for Males ages 10–59 years old and Females 10–19 years old.
**Table S6.** Dynamometry Muscle Strength Results
**Table S7.** Motor Performance Assessments of Definite Adult and Child MM
**Table S8.** Non-ambulatory Dynamometry Muscle Strength Results
**Table S9.** Pearson’s partial correlation between dominant muscle strength controlled for sex
**Table S10.** Motor Performance Assessments in Definite and Unlikely MM
**Table S11.** Pearson’s Correlations
**Table S12.** Summary of data that led to final selection of MM-COAST assessments

Supp Figure 1 **Figure S1.** Dynamometry muscle strength testing revealed symmetric pattern of muscle weakness in MM. Pearson’s correlation of Right: Left was performed. R-values are presented above each muscle group. All muscle groups had significant p-values (*p* < 0.0001). Dots represent outliers. Results indicate significant symmetry in each muscle group tested.

Supp Figure 2 **Figure S2.** Principle component analysis (PCA) of dynamometry-measured muscle strength. PCA indicates that all muscle groups correlate with one another with the exception of neck flexion and pinch.

Supp Figure 3 **Figure S3.** Pearson’s correlation between 6-minute walk test (6MWT) total distance walked and age, height, and weight. 3a-c. Scatter plots indicate lack of interaction between total distance walked and age, height and weight. 3d. Two sample t-test comparison showed no difference in the total distance walked between genders.

Supp Figure Legends

## Figures and Tables

**Table 1:** Participant demographics

Parameter	Definite MM participants (*N* = 59)		Unlikely MM participants (*N* = 32)		*t*-test *p* values

Age at first visit (mean ± SD, range, years)	21.6 ± 13.9 (7.0–64.6)		27.6 ± 19.0 (6.4–65.9)		
Adult participants, number (%)	24 (41%)		18 (56%)		
Paediatric participants, number (%)	35 (59%)		14 (44%)		
Male gender, number (%)	26 (44.1%)		12 (37.5%)		
Weight (mean ± SD, kg)	50.1 ± 22.7		61.4 ± 23.6		0.009
Height (mean ± SD, cm)	151.7 ± 16.9		157.0 ± 18.9		0.178
Body Mass Index (mean ± SD)	Adult	Child	Adult	Child	Adult *p* = 0.89
	24.6 ± 6.5	18.0 ± 4.5	24.4 ± 9.3	22.1 ± 6.4	Child *p* = 0.040
Right hand dominance (%)	84.7		93.8		
Creatine kinase^a^ (IU/L) mean ± SEM (range)	162.3 ± 16.6		90.9 ± 10.0		0.0003
	(26–703)		(30–278)		
	(*n* = 55)		(*n* = 29)		
Venous lactate^b^ (mmol/L) mean ± SEM (range)	2.0 ± 0.2		1.1 ± 0.1		<0.0001
	(0.8–5.7)		(0.5–3.4)		
	(*n* = 54)		(*n* = 26)		
GDF-15^c^ (pg/mL) mean ± SEM (range)	1655 ± 262.2		524.9 ± 50.7		0.0034
	(183–6000)		(277–1119)		
	(*n* = 44)		(*n* = 20)		

a Upper limit of normal for creatine kinase: 370 IU/L

b Reference values for venous lactate: 0.8–2.0 mM

c Reference values for GDF-15: ≤750 pg/mL

## Appendix

**Table:**

Name	Location	Role	Contribution

Jean Flickinger, MPT	Children’s Hospital of Philadelphia, Philadelphia	First author	Contributed to study design, led data collection and assembly, contributed to results interpretation, drafted the manuscript for intellectual content, manuscript review and revisions, and full access to all of the data in the study
Jiaxin Fan, MS	University of Pennsylvania Perelman School of Medicine, Philadelphia	Project biostatistician	Statistical analysis, full access to all of the data in the study, contributed to results interpretation, reviewed and edited manuscript
Amanda Wellik, BS	Children’s Hospital of Philadelphia, Philadelphia	Co-author	Assembly of data, data interpretation, manuscript review and revisions and full access to all of the data in the study
Rebecca Ganetzky, MD	Children’s Hospital of Philadelphia, Philadelphia University of Pennsylvania Perelman School of Medicine, Philadelphia	Co-author	Contributed to data analysis, patient diagnosis, results interpretation, reviewed and edited manuscript
Amy Goldstein, MD	Children’s Hospital of Philadelphia, Philadelphia University of Pennsylvania Perelman School of Medicine, Philadelphia	Co-author	Contributed to data analysis, patient diagnosis, results interpretation, reviewed and edited manuscript
Colleen C. Mauraresku, MS	Children’s Hospital of Philadelphia, Philadelphia	Co-author	Contributed to patient diagnosis, reviewed and edited manuscript
Allan M. Glanzman, DPT	Children’s Hospital of Philadelphia, Philadelphia	Co-author	Contributed to study design, data collection, reviewed and edited manuscript
Elizabeth Balance, DPT	Children’s Hospital of Philadelphia, Philadelphia	Co-author	Contributed to data collection, preparation of figures, reviewed and edited manuscript
Kristin Leonhardt, DPT	Children’s Hospital of Philadelphia, Philadelphia	Co-author	Contributed to data collection, reviewed and edited manuscript
Elizabeth M. McCormick, MS	Children’s Hospital of Philadelphia, Philadelphia	Co-author	Contributed to patient diagnosis and data collection, reviewed and edited manuscript
Brianna Soreth, MA	Children’s Hospital of Philadelphia, Philadelphia	Co-author	Enrolled participants, contributed to data collection, reviewed and edited the manuscript
Sara Nguyen, MPH	Children’s Hospital of Philadelphia, Philadelphia	Co-author	Enrolled participants, contributed to data collection, reviewed and edited manuscript
Jennifer Gornish, BSN	Children’s Hospital of Philadelphia, Philadelphia	Co-author	Contributed to data collection, reviewed and edited manuscript
Ibrahim George-Sankoh, MSc	Children’s Hospital of Philadelphia, Philadelphia	Co-author	Contributed to data collection, results interpretation, reviewed and edited manuscript
James Peterson, MS	Children’s Hospital of Philadelphia, Philadelphia	Co-author	Contributed to patient diagnosis and data collection, reviewed and edited manuscript
Laura MacMullen, BA	Children’s Hospital of Philadelphia, Philadelphia	Co-author	Enrolled participants, contributed to data collection, reviewed and edited the manuscript
Shailee Vishnubhatt, BS	Children’s Hospital of Philadelphia, Philadelphia	Co-author	Contributed to data collection and assembly, results interpretation, manuscript review and revisions
Michael McBride, PhD	Children’s Hospital of Philadelphia, Philadelphia	Co-author	Contributed to data collection reviewed and edited manuscript
Richard Haas, MD	University of California San Diego, La Jolla	Co-author	Contributed to study design, results interpretation, reviewed and edited manuscript
Marni J. Falk, MD	Children’s Hospital of Philadelphia, Philadelphia University of Pennsylvania Perelman School of Medicine, Philadelphia	Co-author	Contributed to study design, obtained study funding, contributed to patient diagnosis, results interpretation, reviewed and edited manuscript
Rui Xiao, PhD	Children’s Hospital of Philadelphia, Philadelphia University of Pennsylvania Perelman School of Medicine, Philadelphia	Lead project biostatistician	Led statistical analysis plan, full access to all of the data in the study, contributed to study design, results interpretation, reviewed and edited manuscript
Zarazuela Zolkipli-Cunningham, MBChB	Children’s Hospital of Philadelphia, Philadelphia University of Pennsylvania Perelman School of Medicine, Philadelphia	Principal investigator, senior and corresponding author	Designed and conceptualized the study, obtained study funding, study investigator, data analysis, data interpretation, manuscript review and revisions, full access to all of the data in the study and final responsibility for the decision to submit the manuscript for publication
