# Supplementary material for: Development of a Mitochondrial Myopathy-Composite Assessment Tool
Source: JCSM Clin Rep. Author manuscript; Available in PMC 2022 Jan 21. (PMC8782422)
Supplement: Supplementary Tables — Table S1. Mitochondrial Myopathy Genetic Etiologies Table S2. Sequence of Study Objective Measures and References Table S3. Assessment Methods of Study Objective Measures Table S4. Clinical Observations, Advantage and Challenges of Study Assessments. Table S5. MM-COAST Composite Score (See Figure 6) Table S5-A. Test scores for Tandem Eyes Open for Males ages 10–59 years old and Females 10–19 years old. Table S6. Dynamometry Muscle Strength Results Table S7. Motor Performance Assessments of Definite Adult and Child MM Table S8. Non-ambulatory Dynamometry Muscle Strength Results Table S9. Pearson’s partial correlation between dominant muscle strength controlled for sex Table S10. Motor Performance Assessments in Definite and Unlikely MM Table S11. Pearson’s Correlations Table S12. Summary of data that led to final selection of MM-COAST assessments [file NIHMS1767033-supplement-Supplementary_Tables.pdf]

Development of a Mitochondrial Myopathy-Composite Assessment Tool (MM-COAST)

Jean Flickinger, MPT[1,2], Jiaxin Fan, PhD[3], Amanda Wellik, BS[1], Rebecca Ganetzky, MD[1,4], Amy Goldstein, MD[1,4], Colleen C. Muraresku, MS[1], Allan M. Glanzman, DPT[2], Elizabeth Ballance, DPT[2], Kristin Leonhardt, DPT[2], Elizabeth M. McCormick, MS[1], Brianna Soreth, MA[1], Sara Nguyen, MPH[1], Jennifer Gornish, BSN[1], Ibrahim George-Sankoh, MSc[1], James Peterson, MS[1], Laura MacMullen, BA[1], Shailee Vishnubhatt, BS[1], Michael McBride, PhD[5], Richard Haas, MD[6,7], Marni J. Falk, MD[1,4], Rui Xiao, PhD[3,4], Zarazuela Zolkipli-Cunningham, MBChB[1,4]*.

Institution: [1]Mitochondrial Medicine Frontier Program, Division of Human Genetics, Children's Hospital of Philadelphia, Philadelphia, PA 19104; [2]Department of Physical Therapy, Children's Hospital of Philadelphia, Philadelphia, PA 19104; [3]Department of Biostatistics, Epidemiology and Informatics, University of Pennsylvania Perelman School of Medicine, Philadelphia, PA 19104, USA; [4]Department of Pediatrics, University of Pennsylvania Perelman School of Medicine, Philadelphia, PA 19104, USA; [5]Cardiovascular Exercise Physiology Laboratory, Division of Cardiology, Children's Hospital of Philadelphia, Philadelphia, PA 19104; [6]Metabolic and Mitochondrial Disease Center and [7]University of California San Diego, La Jolla CA 92093.

*Corresponding Author

Zarazuela Zolkipli-Cunningham, MBChB,

Colket Translational Research Building

Children's Hospital of Philadelphia

Phone: 267 426 4961

Fax: 215 590 0583

zolkipliz@email.chop.edu

Journal submission: Journal of Cachexia, Sarcopenia and Muscle Clinical Reports

Table S1. Mitochondrial Myopathy Genetic Etiologies

Nuclear DNA etiologies (n=18)

Genes	Number (%)	Mutations
POLG	4 (22)	Individual 1: c.2669A>C:p.Asp890Ala [1]<br>Individual 2: c.[2209G>C:p.Gly737Arg];[c.926G>A:p.Arg309His]$^{\wedge}$<br>Individual 3: c.[752C>T:p.Thr251Ile(;)1760C>T:p.Pro587Leu];c.[1880G>A:p.Arg627Gln]$^{\wedge}$<br>Individual 4: c.[2740A>C:p.Thr914Pro]; c.[1399G>A:p.Ala467Thr]$^{\wedge}$
COQ8A	3 (16.6)	Individual 1: c.[811C>T:p.Arg271Cys];c.[655+3A>C]$^{\wedge}$<br>Individual 2: c.[1042C>T:p.Arg348*];c.[830T>C:p.Leu277Pro]$^{\wedge}$<br>Individual 3: c.[1042C>T:p.Arg348*]; c.[830T>C:p.Leu277Pro]$^{\wedge}$
AFG3L2	2 (11)	Individual 1: c.1153G>A:p.Gly385Ser$^{\#}$<br>Individual 2: c.571G>A:p.Val191Ile$^{\&}$
MECR	1 (5.6)	c.[830+2dupT:IVS7+2dupT]; c.[695G>A:p.Gly232Glu]$^{\wedge}$
MPV17	1 (5.6)	c.[191C>T:p.Pro64Leu]; c.375+5G>T$^{\wedge}$
FARS2	1 (5.6)	c.[407C>A:p.Pro136His];partial gene deletion$^{\wedge}$
AIFM1	1 (5.6)	c.720C>T:p.Asp240Asp$^{\#}$[2]
TWINKLE	1 (5.6)	c.1110C>G: p.Phe370Leu$^{\&}$
ECHS1	1 (5.6)	c.[518C>T:p.Ala173Val];c.[123_124delAG:p.Gly42EGlufs*3]$^{\wedge}$
SDHA	1 (5.6)	c.[91C>T:p.Arg31*]; c.[454G>A:p.Glu152Lys]$^{\wedge}$
MTPAP	1 (5.6)	c.[1007G>T:p.Ser336Ile] and c.[1432A>G:p.Asn478Asp]$^{\wedge}$
SSBP1	1 (5.6)	c.79G>A:p.Glu27Lys$^{\#\circ}$[3]

$^{\#}$*de novo*; $^{\wedge}$confirmed *in trans;* $^{\&}$parental samples unavailable; $^{\$}$maternally inherited, °co-existing mtDNA deletion

Mitochondrial DNA etiologies (n=41)

Genes	Number (%)	Mutations and individual heteroplasmy level(s)	
Single large scale mtDNA deletions	13 (31.7)	a. 50%^{M}<br>b. 49%^{M}, 56%^{Bl}<br>c. <10%^{M}<br>d. 56%^{Buc}<br>e. 15%^{Buc}	f. 58%^{Bl}<br>g. 30%^{Bl}<br>h. 26%^{Bl}<br>i. 23%^{Bl}<br>j. Heteroplasmy levels not measured (n=4)
MT-TF	1 (2.4)	m.636A>G<br>a. 56%^{Buc}, 63%^{Bl}	
MT-TL1	7 (17.1)	m.3243A>G<br>a. 67%^{M}<br>b. 45%^{Buc}<br>c. 22%^{Buc}, 12%^{Bl}<br>d. 52%^{Bl}	e. 40%^{Bl}<br>f. 58%^{Bl}, 55%^{Sal}<br>g. 25-50%^{Bl}
	2 (5.0)	m.3288A>G<br>a. 82%^{U}, 46%^{Bl}	

		b. 57%^{Bl}
MT-TK	4 (9.8)	m.8344A>G<br>a. 75%^{Buc} c. 80%^{Bl}<br>b. 88%^{Bl} d. 66%^{Bl}
MT-ATP8	1 (2.4)	Variant pending functional validation, manuscript in progress<br>a. 40%^{Bl}
MT-ATP6	2 (5.0)	m.8993T>G<br>a. 95%^{Buc}, 95%^{Bl}<br>b. 61%^{Buc}
	1 (2.4)	m.8993T>C<br>a. 96%^{U}, 92%^{Bl}
	2 (5.0)	m.9185T>C<br>a. 95%^{Bl}<br>b. 95%^{Bl} (with co-existent m.15243G>A 5%^{Bl}
	1 (2.4)	m.9187T>C<br>a. 80%^{Bl} BL
MT-ND3	4 (9.8)	m.10191T>C<br>a. 83%^{Buc}, 87%^{U}, 84%^{Bl} c. 31%^{Buc}, 3%^{Bl}<br>b. 78%^{Buc}, 44%^{Bl} d. 76%^{Bl}
	1 (2.4)	m.10197G>A<br>a. 90%^{Bl}
MT-ND4	1 (2.4)	m.11778G>A<br>a. 96%^{Bl}
MT-CYB	1 (2.4)	m.15168G>A (heteroplasmy level not measured)

*Heteroplasmy levels in various tissues: Bl= peripheral blood; Buc= Buccal tissue; Sal= Saliva; M= Skeletal Muscle; U= Urine sediment

Table S2. Sequence of Study Objective Measures and References

- All study assessments were conducted from April 2017 through October 2020 at routine clinic visits.
- Participants were tested barefoot without any assistive devices, and followed the same chronological order of assessments, listed below.
- Participants were not allowed to eat immediately prior to the assessments.
- Full assessments were completed within 75-90 minutes.

Domain	Assessment	Age	References for normative data, and results of existing test-retest and inter- and intra-rater reliability testing performed in other disorders.
Muscle Strength	Gross Grasp	6-19<br><br>20+	Grip and pinch strength: norms for 6- to 19-year-olds [4, 5].<br><br>Grip and Pinch Strength: Normative Data for Adults [6].<br><br>Reliability testing:<br>Intra- and Inter-rater reliability testing performed in children and adults with Spinal Muscular Atrophy (SMA) on the preferred side with 20-minute interval between examiners:<br>• Intra-rater reliability:<br>Hand grip intraclass correlation coefficient (ICC): 0.97 ($CI_{95\%}$ =0.93-0.98)<br>• Inter-rater reliability:<br>Hand grip ICC: 0.98 ($CI_{95\%}$ =0.95-0.98) [7]<br><br>Reliability testing:<br>Intra- and Inter-rater reliability in 40 adults with Charcot-Marie-Tooth disease in 6 muscle groups using a hand-held dynamometer.<br>• Intra-rater reliability (one week between tests):<br>Hand grip ICC: 0.98 ($CI_{95\%}$ = 0.96-0.99)<br>• Inter-rater reliability (30 minutes between tests):<br>Hand grip ICC: 0.96 ($CI_{95\%}$ = 0.94-0.98) [8]<br><br>Test-retest reliability:<br>Intersession (administered ≤ one week apart) in healthy female adults:<br>• Pearson product-moment correlation coefficient (mean of 3 trials):<br>Grip strength on the right (R): 0.883 and left (L): 0.929 [9].

Muscle strength	3- point palmar pinch	6-19<br><br>20+	Grip and Pinch Strength: Norms for 6- to 19-Year-Olds [4, 5].<br><br>Grip and Pinch Strength: Normative Data for Adults [6].<br><br>Reliability testing:<br>Intra- and inter-rater reliability in children and adults with SMA tested on preferred side with 20-minute interval between examiners:<br>• Intra-rater reliability: Three-point pinch ICC: 0.94 ($CI_{95\%}$ = 0.89-0.97)<br>• Inter-rater reliability: Three point pinch ICC: 0.92 ($CI_{95\%}$ = 0.84-0.95) [7]<br><br>Reliability testing:<br>Intra- and Inter-rater reliability in 40 adults with Charcot-Marie-Tooth disease in 6 muscle groups using a hand-held dynamometer.<br>• Intra-rater reliability (one week between tests): Three-point pinch ICC: 0.94 ($CI_{95\%}$ = 0.89-0.96)<br>• Inter-rater reliability (30 minutes between tests): Hand grip ICC: 0.95 ($CI_{95\%}$ = 0.90-0.97) [8]<br><br>Test-retest reliability:<br>Inter-session reliability (administered ≤ one week apart) in healthy female adults:<br>• Pearson product-moment correlation coefficient (mean of 3 trials): Palmar pinch: R: 0.812, L: 0.846 [9].
Muscle Strength	Hand-held dynamometry	4-17<br><br>18-65	Reference Values of Maximum Isometric Muscle Force obtained in 270 Children Aged 4–16 Years by Hand-Held Dynamometry [5].<br><br>Muscle Force Measured using "Break" Testing with a Hand-Held Myometer in Normal Subjects Aged 20 to 69 Years [10].<br><br>Reliability testing using J-tech Commander Muscle tester (used in our study):

			• Intra-rater and inter-rater reliability of hand-held dynamometry in lower extremities of healthy adults.<br>○ Intra-rater reliability:<br>ICC for ankle, knee, and hip: 0.94-0.99<br>○ Inter-rater reliability:<br>ICC for ankle, knee, and hip: 0.84-0.90 [11]<br><br>• Concurrent validity of hand-held versus stationary dynamometry in testing shoulder strength.<br>○ Correlation between instruments:<br>Pearson product correlation coefficients:<br>○ Abduction: r=0.81($CI_{95\%}$ =0.66-0.90)<br>○ Flexion: r=0.87 ($CI_{95\%}$ = 0.77-0.93)<br>○ External rotation: r=0.85 ($CI_{95\%}$ = 0.72-0.92) [12]<br><br>Test-retest reliability:<br>Inter-session reliability (administered on 2 consecutive days) in healthy children age matched with children with Duchenne muscular dystrophy (DMD) in 4 muscle groups bilaterally (knee extension, elbow flexion, hip extension, and shoulder abduction):<br><br>• Intersession reliability:<br>Pearson product-moment correlation coefficient:<br>Healthy children: r=0.74-0.99<br>Children with DMD: r=0.83-0.99 [13]<br><br>Reliability testing:<br>Intra-rater and Inter-rater reliability in children and adults with SMA in 6 muscle groups: hand grip, elbow flexion, 3-pt pinch, knee extension, knee flexion, and foot dorsiflexion: on preferred side (20-minute interval between examiners):<br>• Intra-rater reliability:<br>○ Elbow flexion ICC: 0.98 ($CI_{95\%}$ =0.95-0.99)<br>○ Knee extension ICC: 0.93 ($CI_{95\%}$ =0.85-0.96)

			◦ Knee flexion ICC: 0.97 ($CI_{95\%}$ =0.93-0.98)<br>◦ Foot dorsiflexion ICC: 0.91 ($CI_{95\%}$ =0.83-0.95)<br><br>• <u>Inter-rater reliability:</u><br>◦ Elbow flexion ICC: 0.98 ($CI_{95\%}$ =0.96-0.99)<br>◦ Knee extension ICC: 0.88 ($CI_{95\%}$ =0.77-0.94)<br>◦ Knee flexion ICC: 0.95 ($CI_{95\%}$ =0.90-0.97)<br>◦ Foot dorsiflexion ICC: 0.69 ($CI_{95\%}$ =0.46-0.83) [7]<br><br><u>Test-retest reliability:</u><br>Intra-session and inter-session reliability testing in healthy adults in 8 muscle groups included in this study and the MM-COAST. Neck flexors were tested for intersession reliability only.<br><br>• <u>Intra-session reliability:</u><br>ICC (3 trials each) = 0.88-0.99<br><br>• <u>Inter-session reliability:</u><br>ICC (2-week interval): All muscle groups except dorsiflexors: 0.80-0.98<br>Ankle dorsiflexors: 0.68 dominant, 0.535 non-dominant [10].
Muscle Fatigue	Dynamometry repetitions	4-65	Muscle Force Measured using “Break” Testing with a Hand-Held Myometer in Normal Subjects Aged 20 to 69 Years [10].<br><br>This reference provides guidance only for the positions used for testing elbow and hip flexion dynamometry repetitions in the MM-COAST. The protocol for dynamometry repetitions in the MM-COAST is outlined in Table S3.<br><br>This reference also provides test-retest reliability results for dynamometry muscle strength testing using a break test, 3 trials each, with 5 second rests in between. The high ICC reported in this reference [10] indicates the high reliability of repeat muscle testing with a dynamometer in the

			select muscle groups included in this study and our MM-COAST dynamometry repetitions protocol.<br><br>Test-retest reliability:<br>Intra-session and inter-session reliability testing in healthy adults performed in 8 muscle groups, including elbow and hip flexion:<br>• Intra-session reliability (3 trials with 5 second rests between):<br>Dominant elbow flexion: ICC=0.988<br>Dominant hip flexion: ICC= 0.952<br><br>• Inter-session reliability (interval between testing: 2 weeks):<br>Dominant elbow flexion: ICC= 0.934<br>Dominant hip flexion: ICC= 0.905 [10]
Functional Mobility Assessment	North Star Ambulatory Assessment		Reliability of the North Star Ambulatory Assessment in a multicentric setting [14, 15].<br><br>Reliability Testing:<br>Inter-rater reliability between 5 physical therapists rating videos of 3 individuals with Duchenne Muscular Dystrophy (DMD) (n=30), and another group of 17 physical therapists rating videos of 6 individuals with DMD (n=51):<br>• Inter-rater ICC for consistency of total score:<br>o 0.95 (n=30)<br>o 0.95 (n=51)<br>• Inter-rater ICC for absolute agreement of total score:<br>o 0.95 (n=30)<br>o 0.93 (n=51)[15]<br><br>Reliability testing:<br>Intra-rater and inter-rater reliability with 13 physical therapists scoring 3 videos of individuals with DMD:<br><br>• Intra-rater reliability (interval of one month): ICC of total NSAA score = 0.95<br>• Inter-rater reliability of total NSAA score = 0.995 [14]

Balance	Static Balance Assessments:<br><br>Single Leg Stance and Tandem Stance	3-60+	Reference Values for Developing Responsive Functional Outcome Measures Across the Lifespan [16].<br><br><u>Test-retest reliability</u>:<br>Inter-session reliability in children with intellectual disability (administered 2 weeks apart):<br>• <u>Inter-session reliability:</u><br>ICC for BOT 2 balance subtest: 0.99 (95% CI, 0.98-0.99) [17]
Exercise intolerance (Strength-Based)	30 second Sit to Stand	3-60+	Reference Values for Developing Responsive Functional Outcome Measures Across the Lifespan [16].<br><br><u>Test-retest reliability</u>:<br>Inter-session reliability in adults with Parkinson's disease (administered 6-8 days apart):<br><br>• <u>Intersession reliability:</u><br>Intraclass correlation coefficient:<br>$ICC_{(2,2)}$= 0.94 [18, 19]
Dexterity (Finger Dexterity)	Nine Hole Peg Test	3-60+	Reference Values for Developing Responsive Functional Outcome Measures Across the Lifespan [16].<br><br><u>Reliability testing:</u><br>Inter-session (4-6-week interval) and inter-rater (simultaneous scoring by 2 examiners) reliability in elementary school children:<br>• <u>Test-retest</u>:<br>Inter-session correlation coefficient:<br>○ Dominant hand r_{503} = 0.81<br>○ Non-dominant hand r_{503} = 0.79<br><br>• <u>Inter-rater reliability:</u><br>Correlation coefficients between two occupational therapists (OT): r_{416} = 0.99<br><br>Correlation coefficients between one OT and a teacher: r_{106} s = 0.99 [20]<br><br><u>Reliability testing:</u><br>Intra-rater, inter-rater, and test-retest (includes both intrasession and intersession) reliability in a

			review of studies performed with adults with Multiple Sclerosis (MS):<br>• Intra-rater, Inter-rater, and test-retest reliability: range r=0.86-0.98 [21]
Dexterity (In-Hand Manipulation)	Functional Dexterity Test	3-60+	Reference Values for Developing Responsive Functional Outcome Measures Across the Lifespan [16].<br><br>Test-retest reliability:<br>Inter-session in healthy adults (administered twice bilaterally within 48 hours):<br>• Inter-session ICC (for net time): $ICC_{(3,1)}$ =0.94 ($CI_{95\%}$ = 0.93-0.95) [22]<br><br>Reliability testing:<br>Inter-session and inter-rater reliability in typically developing children tested twice bilaterally (mean interval between testing 3.5 days, range 1-27 days):<br><br>• Test-retest reliability: Inter-session $ICC_{(2,1)}$ = 0.90 ($CI_{95\%}$ = 0.783-0.957)<br><br>• Inter-rater reliability (3 examiners scoring simultaneously): ICC= 0.99 (95%CI =0.99-1.0) [23]
Exercise intolerance (Mobility-Based)	6-Minute Walk Test	3-60+	Six Minute Walk Test Demonstrates Motor Fatigue in Spinal Muscular Atrophy [24].<br><br>Reference Values for Developing Responsive Functional Outcome Measures Across the Lifespan [16].<br><br>Test-retest reliability:<br>Intrasession in adults with Alzheimer's disease (30-60-minute rest between tests):<br>• Intrasession $ICC_{(2,1)}$ = 0.987 [25]<br><br>Test-retest reliability:<br>Inter-session reliability in healthy children (mean testing interval was 18 days):

			Inter-session ICC =0.94 ($CI_{95\%}$ 0.89-0.96) [26, 27]

©2020 The Children's Hospital of Philadelphia. All rights reserved.

Table S3. Assessment Methods of Study Objective Measures

Domain/Test	Device(s) Utilized	Position	Procedure
Muscle Strength:<br><br>Gross Grasp and Palmar Pinch Dynamometry	Jamar Smart Hand Dynamometer (Patterson Medical)<br><br>Mechanical Pinch Gauge (PG-30 by B + L Engineering range 0-30 pounds in 1 lb. increments) or a Baseline Mechanical Pinch Gauge (0-10 lb in 0.25 lb. increments)	Both gross grasp and pinch measurements were obtained with the participants in the sitting position with shoulder adducted in neutral rotation, elbow flexed to 90 degrees, and forearm in neutral with wrist between 0-30 degrees extension and 0-15 degrees ulnar deviation.<br><br>Bilateral sides tested. Dominant side was noted based on hand preference as defined by their writing hand.	Gross grasp and palmar (3 point) pinch were measured in all age groups (ages 6-60+) using the standard positioning and normative values [4, 6].<br><br>Three consecutive measurements were obtained on each side. The average of the 3 scores was included in analysis [4, 6]. Each participant was allowed a ~ 5-10 second rest between each measurement.<br><br>Z-score calculation = (Patient value - Normative mean)/Normative standard deviation (SD).
Muscle Strength:<br><br>Upper and Lower Extremity Dynamometry	JTech Commander PowerTrack Muscle Dynamometer	To maintain uniformity in measurements, a standard position was adopted for each muscle group tested [5, 10].<br><br>Testing was performed in the supine position for adults and both supine and sitting positions for children.<br><br>Bilateral sides tested. Dominant side was noted based on hand preference as defined by writing hand.	Across 13 distinct muscle groups listed in Figure 1, 11 muscle groups were measured in adults and 12 in children, in accordance with dynamometry protocols [5, 10]. Shoulder external rotation was measured in adults only, while knee extension and knee flexion were measured in children only.<br><br>To measure maximum voluntary muscle strength, a break test was used with a hand-held dynamometer. Each test was performed by experienced physical therapists who were skilled in the testing procedure and were trained to maintain consistency of technique. Each measurement was performed once, and the physical therapist ensured maximum effort

			was achieved for each muscle group tested. If maximum effort was not achieved, as perceived by the physical therapist, one repeat measurement was allowed to be obtained after a minimum of 15 seconds rest.<br>Data were presented as mean z-scores ± SD based on normative values [5, 10].<br>Participants in the 17 to 19-year-old age range (not included in established protocols [5, 10], followed the following procedure:<br>• 17-year-old participants: Beenakker et al. protocol [5] was applied<br>• 18 to 19-year-old participants: Phillips et al. protocol [10] was applied<br><br>Z-score calculation = (Patient value - Normative mean)/Normative SD.
Muscle Fatigue:<br><br>Dynamometry Repetitions	JTech Commander PowerTrack Muscle Dynamometer<br><br>½ inch yoga mat	Standard position was adopted for testing of the elbow and hip flexors [10] for all ages in supine position on a plinth covered with a ½ inch thick yoga mat (to prevent sliding).<br><br>Testing for elbow flexors was performed with the shoulder abducted to 10 degrees, elbow flexed to 90 degrees and the forearm in supinated position.<br><br>Testing for the hip flexors was performed with the hip and knee flexed to 90 degrees with	Yoga mat was placed on plinth to prevent sliding of patient which could occur with smaller children and adults, or very strong participants.<br><br>Muscle repetitions were tested on one side for elbow and hip flexors for six consecutive repetitions with 1-2 second rest between each repetition, which is the length of time to return to the start position. The same protocol was repeated on the opposite side.<br><br>Negative percent decrement was calculated from the 6th to 1st repetition using the raw dynamometry data (lbs.) with the formula: [(6th repetition -1st repetition)/ 1st repetition] x 100.

		the lower leg supported by the physical therapist.<br><br>Bilateral sides tested	Results can also be expressed as the decline in mean muscle strength z-scores ± SD from the 1st to the 6th repetition.
Functional Mobility:<br><br>North Star Ambulatory Assessment	A Fitness Aerobic Step with one riser secured (29” width, 6” height)<br><br>Kaye adjustable bench<br><br>Airex exercise mat<br><br>Stopwatch	Kaye adjustable bench was used to achieve a 90-degree hip and knee position for stand up from chair activity.	The NSAA consists of 17 functional activities [14]. For each activity, a score of 2 (achieving fully), 1 (achieving with assistance or some degree of compensation), or a 0 (unable to complete task) was recorded and a total score was recorded out of a possible 34 points [14]. A Fitness Aerobic Step was used for the climbing up and down box step items and a Kaye adjustable bench for the stand up from chair activity. An Airex exercise mat was used for the rise from the floor item which was timed with a stopwatch. In this study, the 10 meter run was graded only and was not timed.
Balance:<br><br>Static Balance Assessments	Line 2” width on floor<br><br>Target (from Bruininks-Oseretsky Test of Motor Proficiency Second Edition (BOT-2) [17]	Participants were positioned on a line 5 meters away from the target placed at eye level, standing in the following balance positions sequentially: 1) Single leg stance eyes open was assessed clinically only to screen for the ability to proceed to single leg stance with eyes closed. The single leg stance eyes open position was not included in our data analysis nor in the MM-COAST composite score as this was not part of the balance protocol) [16, 28]; and 2)	All balance testing was performed without shoes or socks.<br><br>Balance testing was performed on the dominant leg only. The dominant leg was determined by the leg that the participant reported they would kick a ball with and often matched the dominant upper extremity.<br><br>Participants were asked to hold each position for up to 10 seconds for children and up to 20 seconds for adults [16, 28]. The timer was stopped when the participant stepped off the line or could not maintain position per protocol, or patient achieved the target goal of 10 or 20

		Tandem stance eyes open and eyes closed.<br><br>Single leg stance was performed on a line with hands on hips standing on dominant leg, with opposite leg bent with knee flexed to 90 degrees and legs kept apart with head and trunk upright with eyes open, focused on target, followed by the eyes closed position.<br><br>Tandem stance was performed starting with the dominant foot on a line, with hands on hips, then placing opposite foot in front of dominant foot (heel of non-dominant foot touching toes of dominant foot) with head and trunk upright, eyes open, focused on target, followed by eyes closed position.	seconds depending on age. Short rests of <10 seconds between each position was allowed.<br><br>For eyes closed positions, participants were directed to attain the position first with eyes open, focused on target, then once stable, they could close their eyes. Timer was started once eyes were closed.<br><br>If an individual was able to stand independently, and balance assessment was attempted, but imbalance was so severe that the individual was unable to attain or maintain a balance testing position in the protocol [16], they received a raw score of 0 seconds for that position.<br><br>Individuals who were unable to stand independently were not assessed for balance tests (not tested, NT).<br><br>Z-score calculation = (Patient value - Normative mean)/Normative SD.
Exercise Intolerance (strength-based assessment):<br><br>30 second Sit to Stand Test	Kaye adjustable bench<br><br>Stopwatch	The 30 second Sit to Stand test was performed with the participant sitting on a bench adjusted so that the participant's hip and knee flexion angles were at 90 degrees (90/90) with arms	Once positioned correctly in the seated position, the participant was instructed to "stand all the way up" and "sit all the way down" as quickly as they could until they were asked to stop. Repetitions were counted only if the knees and hips were fully extended upon standing. The final repetition was counted provided the participant attained standing

		crossed at the chest and feet on the floor.	upright fully before 30 seconds was reached. The number of complete ‘sit to stands’ were recorded in 30 seconds [16].<br><br>Z-score calculation = (Patient value - Normative mean)/Normative SD.
Dexterity:<br><br>Nine Hole Peg Test (9HPT) and Functional Dexterity Test (FDT)	Jamar 9-Hole Peg Test<br><br>FDT hardwood pegboard<br><br>Stopwatch<br><br>Table<br><br>Footrest (if needed for smaller children)	The 9 hole peg test (9HPT) followed by the Functional Dexterity Test (FDT) were completed on the dominant side only, with the participant seated comfortably with feet supported on the floor (or on a footrest if needed for smaller children and adults) and table at elbow height.<br><br>The 9HPT board was positioned with the container for the pegs on the same side as the dominant side being tested with the non-dominant hand supporting the opposite side of the board.	The participant was timed using a stopwatch after directions and demonstrations were given.<br>• 9HPT: the participant was instructed to place 9 pegs, one at a time into the holes then subsequently return all the pegs back into the dish one at a time, as fast as they could. The timer was started as soon as the first peg was touched and was stopped when the last peg landed in the collection container [16].<br>• FDT: the participant was instructed to flip the pegs over to the opposite side as quickly as they could while avoiding compensations including use of the board or other part of the body to flip the peg or supinating the forearm past neutral to turn the peg in the palm of the hand. If a compensation was noted, the timer was stopped, the peg was placed back in the board to re-attempt, and the timer was continued once the participant touched the peg to continue. Total time to complete each test was recorded in seconds and data analysis was performed using normative values [16].

			Z-score calculation = (Normative value - patient mean)/Normative SD. This is the reverse calculation compared to the other domains, as a lower patient measurement reflects better ability.
Exercise intolerance (mobility-based assessment): 6-Minute Walk Test minute distances	Standard 6-minute walk test course<br><br>2 cones	Cones were placed 25 meters apart in a long, quiet hallway with each meter marked along the course in the center.	The 6-minute walk test was completed as the final assessment after a 10-minute rest was provided. The participant was instructed to walk around the cones as fast as they could for six minutes but was not allowed to run. Verbal cues were limited to announcing each minute until 6 minutes was reached and the participant was advised to stop [16, 24]. The time was noted each time the participant rounded the cone.<br><br>Distance walked was recorded each minute as well as the total distance covered in 6 minutes and can be presented as raw data (meters), z-scores and/or minute distance slope analysis. In addition, 6-minute walk test was also expressed as percent (%) predicted in this study, based on population normative values [16].<br><br>Z-score calculation = (Patient value - Normative mean)/Normative SD.<br><br>Percent (%) predicted = (Patient Value/Predicted Value)*100

©2020 The Children's Hospital of Philadelphia. All rights reserved.

Table S4. Clinical Observations, Advantage and Challenges of Study Assessments.				
Domain	Test	Clinical Observations	Advantages of Assessment	Observed Technical Challenges
Muscle Strength	Dynamometry of Upper and Lower Extremities	Dynamometry [4-6, 10] was accurately administered in all participants ≥ 6 years where cognition did not interfere with a valid test. In non-ambulatory participants, dynamometry was performed in muscle groups where the position per protocol was able to be maintained. Total testing time was ~ 20-25 minutes.	More precise, objective measure of strength than manual muscle testing by Medical Research Council (MRC) grading.<br><br>Able to test in non-ambulatory participants (unless movement is limited by severe muscle weakness or contractures).	Impaired cognition may limit ability to achieve a reliable assessment.<br><br>Limited normative data across the lifespan available for specific age ranges including 17- to 19-year-old individuals.<br><br>Other published normative data can be applied to this method, however the positions of testing need to be exactly the same.<br><br>Broad age groups through the age span is associated with larger standard deviations [5, 29].<br><br>Most protocols separate children and adults, which requires different testing positions in children compared to adults [5, 10, 30] .
Muscle Fatigue	Dynamometry repetitions for Elbow and Hip Flexion	Total testing time for both sides was ~ 10 minutes.	Tests muscle-specific fatigue	Difficult to perform if cognitively impaired or if poor motor control impacts timing of muscle contractions.

				In terms of feasibility across all age groups, the hip flexion test position may pose challenges for some children and adults as the test leg is supported by the physical therapist (PT)'s arm (compared to the stable support surface when testing elbow). Care should be taken by the PT to avoid subtle shifts in arm position allowing hip rotation and confusing cues to the patient.
Balance	Single Leg Eyes Closed<br><br>Tandem Stance Eyes Open and Eyes Closed	Balance testing was feasible in participants of all ages. If participants were too weak or unable to stand independently, we did not pursue balance assessments.<br><br>Total testing time was ~10 minutes.	Quick assessments of static balance, well tolerated	Some participants reported fatigue on the tested dominant side.<br><br>Ceiling effect noted, where many children up to age 19 years were able to hold balance assessment positions well past the 10 second stopping point per protocol [16].<br><br>Individuals who are unable to stand independently are not able to complete this assessment.
Exercise Intolerance	Strength-based:<br>30 second Sit to Stand	Feasible assessment for all ages	Quick test of exercise intolerance and strength-based functional activity.	If a participant is not able to stand up from sitting without use of their arms, usually due to weakness or imbalance,

			Assessment involves short bursts of high intensity exercise, unlike the 6-minute walk test.<br><br>This test is better suited to cognitively impaired individuals who are unable to reliably perform the 6-minute walk test.<br><br>Requires minimal space and equipment to complete.	they are not able to complete this assessment.
	Mobility-based:<br>6-minute walk test	Feasible for all ages	Mobility based test of exercise intolerance.<br><br>The ability to ambulate is clinically meaningful to participants.	If a participant's cognition or balance is significantly limited, this may impede full effort, leading to inability to complete a valid assessment.
Dexterity	Nine Hole Peg test	Testing time was ~ 5 minutes	Feasible and quick assessment.<br><br>Nine Hole Peg Test (9HPT) is easier performed by younger participants, and those with mild cognitive delay.	Does not measure in-hand manipulation skills, therefore more subtle fine motor dexterity deficits may be missed if not performed in conjunction with the Functional Dexterity Test (FDT).
	Functional Dexterity Test	Testing time was ~ 5 minutes	Feasible and quick assessment.<br><br>Valid test of in-hand manipulation which	Participants with significant deficits in fine motor coordination are unable to complete test as

			correlates to functional tasks that require in-hand manipulation and a palmar pinch prehension pattern such as buttoning, tying shoe laces, screwing a nut and bolt, and writing [31-33].<br><br>Identifies more subtle deficits in participants who may score in the normal range when tested on 9HPT or other simpler grasp and release dexterity instruments [34].	compensatory movement patterns are not allowed.<br><br>Test directions for FDT can be more difficult to follow for cognitively impaired participants compared to 9HPT directions.

©2020 The Children's Hospital of Philadelphia. All rights reserved.

Table S5. MM-COAST Composite Score (See Figure 6)	
Step 1:	Determine dominant side z-score (or percent decrement for dynamometry repetitions in muscle fatigue domain) for each assessment raw score using formulas in Table S3 and published normative data for each assessment [4-6, 10, 16].
Step 2:	Assign a test score ranging from -3 to +3 for each assessment in each domain based on the z-score (Chart A), or percent decrement for dynamometry repetitions in the muscle fatigue domain (Chart B)
Step 3:	Sum up the test scores from Step 2 and divide by the number of tests completed in each domain to achieve the Domain score)
Step 4:	Sum up Domain scores from Step 3.<br>Note: ambulatory participants must complete at least 3 domains and non-ambulatory participants must complete at least 2 domains. See scoring considerations below.
Step 5:	Divide Sum of Domain Scores from Step 4 by the number of completed Domains to achieve the **MM-COAST Composite Score**
Scoring considerations:	
1)	The 6-Minute Walk Test assessment of exercise intolerance can be analyzed both as a z-score (incorporated in the composite score) and by linear mixed effects model for the minute distance slope (not incorporated in the composite score). Minute distance slope analysis does not require normative data but can only be analyzed at the cohort-level and not at the individual-level.
2)	In some cases, a participant may not be able to complete all 5 domains due to patient refusal or various physical limitations imposed by the myopathy. In order to ensure that MM COAST Composite Scores are fully reflective of all MM key domains, we recommend that ambulatory individuals should complete assessments in a minimum of 3/5 MM-COAST domains, while non-ambulatory individuals should complete a minimum of 2/3 domains (muscle strength, muscle fatigue and dexterity assessments) to achieve a truly representative MM COAST Composite Score.
3)	If a participant is not able to complete an assessment in a specific domain, a test score should not be assigned. For example, if only 3 of the muscle groups were tested, the sum of the test scores of the 3 muscles assessed would be divided by 3 (Step 3 above).<br>Similarly, if an entire domain was not completed such as elbow flexion for muscle fatigue, then that domain is not considered a completed Domain in the equation in Step 5 above.

4)	Special considerations:<br>1. If an individual is not able to complete the Functional Dexterity Test (FDT) due to observed significant in-hand manipulation deficits and an attempt was made to complete the assessment, the maximum test score of 3 may be assigned for the FDT in the Dexterity domain in Step 2 above. Similarly, a test score of 3 should be assigned for a failed Nine Hole Peg Test (9HPT) in the Dexterity domain in Step 2 above if the reason for failure is due to observed significant finger dexterity deficits. These observations should be made by an experienced evaluator (physical or occupational therapist).<br>2. If a test in any domain is not attempted due to reasons that include patient refusal, difficulty following commands and poor cognition, this is considered "not tested" (NT) and no test score is assigned.<br>3. If an individual is not able to stand independently for balance assessments, 30 second sit to stand (30s STS) and 6-minute walk test (6MWT), they should not be scored and considered NT with no test score assigned.<br>4. Specific considerations for balance assessments:<br>• For individuals in these age groups: males 10-59 years old and females 10-19 years old, the normative data used in this study [16] lists a standard deviation of 0 for Tandem Stance Eyes Open which precludes a z-score calculation.<br>• In order to ensure accurate representation of imbalance in the MM-COAST Composite Score in these age groups, we devised a test scoring approach (Table S5-A) to ensure standardized assignment of test scores (Step 2 above) based on the measured Tandem Stance Eyes Open assessment time (raw score).

Table S5-A. Test scores for Tandem Eyes Open for Males ages 10-59 years old and Females 10-19 years old.

Measured Time (seconds)	Approximate SD	Assigned score
>=20	>=3.1	-3
16-19	2.1 to 3	-2
11-15	0.1 to 2	-1
9-10	0 to -2	0
4-8	-2.1 to -3	1
2-3	-3.1 to -4	2
0-1	<=-4.1	3

©2020 The Children's Hospital of Philadelphia. All rights reserved

Table S6. Dynamometry Muscle Strength Results		
**Hand-Held Dynamometry** mean z-scores ± SD (95% CI)	**Definite (n=59)**	
	**Dominant**	**Non-Dominant**
Neck Flexion	-1.2 ± 1.8 (-1.84 – -0.60)	
Shoulder Abduction	-1.7 ± 1.6 (-2.10 – -1.22)	-1.8 ± 1.7 (-2.21 – -1.32)
Shoulder External Rotation[a]	-1.8 ± 2.0 (-2.74 – -0.87)	-2.0 ± 2.0 (-3.01 – -1.06)
Elbow Extension	-0.9 ± 1.7 (-1.31 – -0.42)	-0.9 ± 1.7 (-1.34 – -0.43)
Elbow Flexion	-2.6 ± 2.1 (-3.19 – -2.08)	-2.3 ± 1.9 (-2.83 – -1.82)
Wrist Extension	-3.4 ± 1.7 (-3.85 – -2.90)	-3.6 ± 1.9 (-4.08 – -3.06)
Gross Grasp	-2.1 ± 1.1 (-2.38 – -1.73)	-2.0 ± 1.4 (-2.39 – -1.62)
Pinch Strength	-2.5 ± 2.8 (-3.47 – -1.49)	-3.0 ± 1.4 (-3.49 – -2.50)
Hip Flexion	-2.5 ± 2.3 (-3.15 – -1.87)	-2.4 ± 2.1 (-3.04 – -1.81)
Hip Abduction	-1.5 ± 1.9 (-1.98 – -0.97)	-1.7 ± 1.8 (-2.19 – -1.22)
Knee Extension[b]	-1.7 ± 1.4 (-2.23 – -1.22)	-2.0 ± 1.2 (-2.43 – -1.59)
Knee Flexion[b]	-2.8 ± 1.3 (-3.22 – -2.30)	-2.8 ± 1.3 (-3.27 – -2.35)
Ankle Dorsiflexion	-2.4 ± 2.5 (-3.07 – -1.69)	-2.2 ± 2.3 (-2.86 – -1.52)
**Comparison of UE proximal and distal muscle groups**	**Weakest Dominant Proximal muscle group**	**Weakest Dominant Distal muscle group**

Muscle Group (mean ± SD)	Elbow Flexion -2.6 ± 2.1	Wrist Extension -3.4 ± 1.7
T-test *p*-value comparison of elbow flexion to wrist extension	0.018	
**Comparison of LE proximal and distal muscle groups**	**Weakest Dominant Proximal muscle group**	**Weakest Dominant Distal muscle group**
Muscle Group (mean ± SD)	Hip Flexion -2.5 ± 2.3	Ankle Dorsiflexion -2.4 ± 2.5
T-test *p*-value* comparison of hip flexion to ankle dorsiflexion	0.40	

[a] Testing performed in Adult MM group, not tested in child cohort per protocol [10].

[b] Testing performed in Child MM group, not tested in adult cohort per protocol [5].

Table S7. Motor Performance Assessments of Definite Adult and Child MM

**Hand-Held Dynamometry** mean z-scores ± SD (95% CI)	**Adult (n=24)**		**Child (n= 35)**		**t-test *p*-value[bc]**
	Dominant	Non-Dominant	Dominant	Non-dominant	
Neck Flexion	-1.2 ± 2.3 (-2.44 – -0.02)		-1.2 ± 1.3 (-1.84 – -0.58)		0.75
Shoulder Abduction	-1.3 ± 2.1 (-2.29 – -0.40)	-1.8 ± 1.9 (-2.61 – -0.89)	-1.9 ± 1.2 (-2.28 – -1.45)	-1.8 ± 1.5 (-2.29 – -1.25)	0.71
Shoulder External Rotation[#]	-1.8 ± 2.0 (-2.74 – -0.87)	-2.0 ± 2.0 (-3.01 – -1.06)	-	-	
Elbow extension	-1.4 ± 1.9 (-2.23 – -0.62)	-1.5 ± 2.0 (-2.34 – -0.64)	-0.5 ± 1.4 (-0.98 – 0.02)	-0.5 ± 1.4 (-0.98 – 0.01)	0.008
Elbow flexion	-3.2 ± 3.0 (-4.44 – -1.92)	-2.3 ± 2.6 (-3.47 – -1.10)	-2.3 ± 1.1 (-2.63 – -1.87)	-2.4 ± 1.4 (-2.82 – -1.88)	0.19
Wrist Extension	-4.6 ± 1.4 (-5.23 – -3.91)	-4.8 ± 1.8 (-5.60 – -4.00)	-2.6 ± 1.4 (-3.07 – -2.07)	-2.8 ± 1.5 (-3.31 – -2.27)	<0.0001
Gross Grasp	-1.9 ± 1.1 (-2.41 – -1.42)	-1.7 ± 1.4 (-2.38 – -1.07)	-2.2 ± 1.2 (-2.60 – -1.70)	-2.2 ± 1.3 (-2.68 – -1.70)	0.36
Pinch strength	-2.0 ± 1.2 (-3.48 – -0.48)	-1.9 ± 1.0 (-3.20 – -0.64)	-2.6 ± 3.0 (-3.73 – -1.40)	-3.2 ± 1.4 (-3.72 – -2.65)	0.23
Hip Flexion	-1.9 ± 3.0 (-3.24 – -0.59)	-1.6 ± 2.6 (-2.82 – -0.36)	-3.0 ± 1.4 (-3.49 – -2.43)	-3.0 ± 1.5 (-3.59 – -2.42)	0.16
Hip Abduction	-1.3 ± 2.6 (2.47 – -0.20)	-1.8 ± 2.5 (-2.88 – -0.68)	-1.6 ± 1.2 (-2.00 – -1.14)	-1.7 ± 1.1 (-2.05 – -1.26)	0.65
Knee Extension[a]	-	-	-1.7 ± 1.4 (-2.23 – -1.22)	-2.0 ± 1.2 (-2.43 – -1.59)	
Knee Flexion[a]	-	-	-2.8 ± 1.3 (-3.22 – -2.30)	-2.8 ± 1.3 (-3.27– -2.35)	
Ankle Dorsiflexion	-3.8 ± 2.6 (-4.87 – -2.63)	-3.2 ± 2.5 (-4.31 – -2.06)	-1.3 ± 1.7 (-1.90 – -0.60)	-1.4 ± 1.7 (-2.05 – -0.65)	0.0004

**Balance Assessment**			
**Balance Test** mean z-scores ± SD (95% CI)	**Adult (n=28)**	**Child (n=25)**	**t-test *p*-value[c]**
Tandem Stance Eyes Open	-5.8 ± 11.6 (-10.26 – -1.28)	-0.4 ± 1.0 (-0.81 – 0.05)	0.033
Tandem Stance Eyes Closed	-2.0 ± 1.7 (-2.67 – -1.37)	-3.2 ± 3.4 (-4.67 – -1.80) (n=24)	0.31
Single Leg Stance Eyes Closed	-1.3 ± 1.0 (-1.74 – -0.96)	-2.0 ± 1.6 (-2.65 – -1.35)	0.10
**Fine Motor Dexterity**			
**Functional Dexterity Test** mean z-scores ± SD (95% CI)	**Adult (n=22)**	**Child (n=21)**	**t-test *p*-value[c]**
	-7.3 ± 7.3 (-10.51 – -4.10)	-4.6 ± 4.3 (-6.54 – -2.64)	0.18
**Nine Hole Peg Test** mean z-scores ± SD (95% CI)	**Adult (n=24)**	**Child (n=29)**	**t-test *p*-value[c]**
	-10.8 ± 14.3 (-16.82 – -4.78)	-6.2 ± 7.4 (-9.00 – -3.37)	0.47
**Exercise Intolerance Assessments**			
**30 second Sit to Stand (30s STS) Test** mean z-scores ± SD (95% CI)	**Adult (n=16)**	**Child (n=22)**	**t-test *p*-value[c]**
	-2.1 ± 0.7 (-2.48 – -1.77)	-2.0 ± 0.8 (-2.33 – -1.59)	0.78
**6-Minute Walk Test minute distance**	**Adult (n=20)**	**Child (n=26)**	

mean ± SEM (meters) (95% CI)					
1 min distance	70.1 ± 3.7 (62.35 – 77.85)		77.4 ± 3.4 (70.38 – 84.31)		
2 min distance	69.0 ± 3.7 (61.17 – 76.83)		74.6 ± 3.2 (68.00 – 81.23)		
3 min distance	69.4 ± 4.5 (59.95 – 78.85)		75.2 ± 3.2 (68.74 – 81.72)		
4 min distance	67.5 ± 3.9 (59.25 – 75.75)		69.8 ± 2.9 (63.89 – 75.72)		
5 min distance	67.7 ± 3.7 (59.99 – 75.41)		70.3 ± 3.1 (63.94 – 76.60)		
6 min distance	66.9 ± 3.8 (58.82 – 74.88)		69.4 ± 4.2 (60.68 – 78.09)		
Total distance	410.6 ± 21.8 (364.90 – 456.20)		436.7 ± 17.6 (400.30 – 473.00)		0.29
Total distance z-score Mean ± SD (95% CI)	-3.4 ± 1.4 (-4.04 – -2.73)		-2.6 ± 1.2 (-3.07 – -2.10)		0.047
	Predicted Total Distance ± SEM (95% CI)	Mean % predicted ± SEM (95% CI)	Predicted Total Distance ± SEM (95% CI)	Mean % predicted ± SEM (95% CI)	0.23
	687.5 ± 13.9 (658.40 – 716.60)	60.4 ± 3.6% (52.86 – 67.91)	660.5 ± 13.0 (633.70 – 687.30)	66.1 ± 2.3% (61.30 – 70.79)	
**Dynamometry Repetitions** mean z-scores ± SD (95% CI)	**Adult (n=11)**		**Child (n=14)**		**t-test *p*-value[bc]**
	Dominant	Non-Dominant	Dominant	Non-dominant	
Elbow Flexion- 1st Repetition	-1.7 ± 2.6 (-3.71 – 0.32) n=9	-1.1 ± 2.3 (-2.93 – 0.65) n=9	-2.0 ± 1.0 (-2.51 – -1.34) n=13	-2.0 ± 1.4 (-2.88 – -1.19) n=13	

Elbow Flexion- 6th Repetition	-2.6 ± 2.5 (-4.50 – -0.73) n=9	-2.0 ± 2.4 (-3.78 – -0.13) n=9	-2.6 ± 0.9 (-3.18 – -2.01) n=12	-2.8 ± 0.9 (-3.39 – -2.23) n=12	
Negative Percent Decrement (%) (mean ± SEM) (95% CI)	-13.9 ± 3.0% (-20.74 – -6.97) n=9	-13.3 ± 4.8% (-24.39 – -2.30) n=9	-15.39 ± 3.2% (-22.43 – -8.36) n=12	-16.9 ± 5.6% (-29.13 – -4.57) n=12	0.57 DOM
Hip Flexion- 1st Repetition	-1.4 ± 2.8 (-3.25 – 0.52) n=11	-1.5 ± 2.4 (-3.35 – 0.28) n=9	-2.1 ± 0.9 (-2.62 – -1.58) n=14	-2.2 ± 1.0 (-2.80 – -1.62) n=14	
Hip Flexion- 6th Repetition	-1.7 ± 2.5 (-3.84 – 0.41) n=8	-2.1 ± 2.6 (-4.48 – 0.34) n=7	-2.6 ± 1.0 (-3.23 – -1.94) n=11	-2.4 ± 1.0 (-3.02 – -1.73) n=11	
Negative Percent Decrement (%) (mean ± SEM) (95% CI)	-8.3 ± 7.6% (-26.29 – 9.7) n=8	-16.3 ± 6.2% (-30.50 – -1.11) n=7	-12.7 ± 5.5% (-25.04 – -0.43) n=11	-8.5 ± 6.9% (-23.96 – 6.91) n=11	0.62 DOM
**Functional Assessment**					
**North Star Scores** mean ± SEM (95% CI)	**Adult (n=26)**		**Child (n=32)**		**t-test *p*-value[c]**
	23.7 ± 1.9 (19.88 – 27.58)		26.3 ± 1.7 (22.81 – 29.69)		0.088

[a] Testing was not performed in adults per protocol [10]

[b] t-test comparison of dominant sides only

[c] t-test comparisons of MM Adults to MM Child participants

Table S8. Non-ambulatory Dynamometry Muscle Strength Results

**Hand-Held Dynamometry** mean z-scores ± SD (95% CI)	**Non-Ambulatory (n=6)**	**Ambulatory (n=53)**	**t-test p-values[b]**
Dominant Elbow Flexion	-3.9 ± 2.5<br>(-6.43 – -1.29)<br>n=6	-2.5 ± 2.1<br>(-3.07 – -1.92)<br>n=52	0.09
Dominant Wrist Extension	-4.6 ± 1.3<br>(-6.73 – -2.47)<br>n=4	-3.3 ± 1.7<br>(-3.77 – -2.78)<br>n=48	0.14
Dominant Hip Flexion	-4.3 ± 1.5<br>(-6.61 – -1.89)<br>n=4	-2.4 ± 2.3<br>(-3.03 – -1.70)<br>n=47	0.041
Dominant Ankle Dorsiflexion	-4.7 ± 3.3<br>(-12.95 – 3.61)<br>n=3	-2.2 ± 2.4<br>(-2.92 – -1.55)<br>n=48	0.16
**Composite Score[a]**<br>Mean score ± SEM (95%CI)	2.7 ± 0.17<br>(2.11 – 3.18)<br>n=4	1.2 ± 0.1<br>(0.94 – 1.37)<br>n=49	0.0001

[a]Non-ambulatory participants must complete at least 2/3 domains to receive a composite score, ambulatory participants must complete at least 3/5 domains to receive a composite score.

[b] t-test comparison of non-ambulatory to ambulatory participants

Table S9. Pearson's partial correlation between dominant muscle strength controlled for sex

**Dominant Muscle Strength**	Age			Height			Weight		
	Number (n)	r	*p*-value	Number (n)	r	*p*-value	Number (n)	r	*p*-value
Neck flexion	34	0.08	0.671	34	0.45	0.009	34	0.45	0.002
Shoulder Abduction	56	0.57	<0.0001	55	0.71	<0.0001	56	0.71	<0.0001
Shoulder External Rotation	20	-0.01	0.708	20	0.28	0.248	20	0.28	0.008
Elbow extension	56	0.36	0.008	55	0.63	<0.0001	56	0.63	<0.0001
Elbow flexion	58	0.34	0.009	57	0.56	<0.0001	58	0.56	<0.0001
Wrist Extension	52	0.52	0.0001	51	0.66	<0.0001	52	0.66	<0.0001
Gross Grasp	53	0.64	<0.0001	53	0.70	<0.0001	53	0.70	<0.0001
Pinch strength	33	0.36	0.040	33	0.27	0.132	33	0.27	0.120
Hip Flexion	49	0.52	0.0001	49	0.62	<0.0001	49	0.62	<0.0001
Hip Abduction	54	0.48	0.0003	53	0.64	<0.0001	54	0.64	<0.0001
Knee Extension	33	0.39	0.029	32	0.65	0.0001	33	0.65	<0.0001
Knee Flexion	33	0.29	0.107	32	0.67	<0.0001	33	0.67	0.001
Ankle Dorsiflexion	50	0.36	0.012	50	0.33	0.021	50	0.33	0.001

Table S10. Motor Performance Assessments in Definite and Unlikely MM			
**Balance**			
**Balance Test (dominant leg)** mean z-scores ± SD (95% CI)	**Definite (n=53)**	**Unlikely (n=32)**	**t-test *p*-values[c]**
Tandem Stance Eyes Open	-3.2 ± 8.8 (-5.65 – -0.80)	-0.6 ± 6.1 (-2.76 – 1.67)	0.021
Tandem Stance Eyes Closed	-2.6 ± 2.7 (-3.32 – -1.84) (n=52)	-0.5 ± 1.4 (-1.01 – -0.01)	<0.0001
Single Leg Stance Eyes Closed	-1.7 ± 1.3 (-2.02 – -1.29)	-0.5 ± 1.0 (-0.82 – -0.08)	<0.0001
**Fine Motor Dexterity**			
**Functional Dexterity Test (dominant side)** mean z-scores ± SD (95% CI)	**Definite (n=44)**	**Unlikely (n=25)**	**t-test *p*-values[c]**
	-5.9 ± 6.0 (-7.70 – -4.03)	-3.1 ± 3.4 (-4.45 – -1.68)	0.033
**Nine Hole Peg Test (dominant side) mean z-scores ± SD (95% CI)**	**Definite (n=53)**	**Unlikely (n=26)**	
	-8.3 ± 11.2 (-11.35– -5.19)	-2.9 ± 3.9 (-4.49 – -1.30)	0.006
**Exercise Intolerance Assessments**			
**30 second Sit to Stand Test** mean z-scores ± SD (95% CI)	**Definite (n=38)**	**Unlikely (n=17)**	**t-test *p*-values[c]**
	-2.0 ± 0.8	-1.7 ± 0.9	0.14

	(-2.28 – -1.78)		(-2.18 – -1.20)		
**6-Minute Walk Test minute distance** mean ± SEM (meters)	**Definite (n=46)**		**Unlikely (n=28)**		**t-test *p*-values[c]**
1 min distance	74.2 ± 2.5		86.7 ± 4.5		
2 min distance	72.2 ± 2.5		85.3 ± 4.2		
3 min distance	72.7 ± 2.7		80.6 ± 4.0		
4 min distance	68.8 ± 2.3		80.6 ± 4.1		
5 min distance	69.2 ± 2.3		81.6 ± 4.3		
6 min distance	68.3 ± 2.9		79.6 ± 4.6		
Total distance walked	425.3 ± 13.7		494.4 ± 24.4		0.011
Total distance z-score Mean ± SD	-2.9 ± 1.3		-1.8 ± 1.6		0.001
Percent predicted walked	Predicted Total Distance ± SEM	Mean % predicted ± SEM	Predicted Total Distance ±SEM	Mean % predicted ±SEM	
	672.2 ± 9.6	63.6 ± 2.1%	667.8 ± 15.2	74.5 ± 3.7%	0.012
Mixed Effects Model	**Definite (n=46)**		**Unlikely (n=28)**		
	Minute Distance Slope	*p*-value	Minute Distance Slope	*p*-value	
	-0.9	0.03	-1.39	0.70	
**Muscle Fatigue**					
**Dynamometry Repetitions** mean z-scores ± SD (95% CI)	**Definite MM**		**Unlikely**		**t-test *p*-values[c]**
	Dominant	Non- Dominant	Dominant	Non- Dominant	
Elbow Flexion- 1st Repetition z-score	-1.8 ± 1.8 (-2.62 – -1.04) n= 22	-1.7 ± 1.8 (-2.49 – -0.85) n= 22	-2.6 ± 2.4 (-4.59 – -0.50) n=8	-2.6 ± 2.7 (-4.87 – -0.30) n=8	

Elbow Flexion- 6th Repetition z-score	-2.6 ± 1.7 (-3.38 – -1.83) n= 21	-2.4 ± 1.7 (-3.22 – -1.67) n=21	-3.6 ± 2.2 (-5.44– -1.73) n=8	-3.8 ± 2.7 (-6.09 – -1.56) n=8	
Negative Percent Decrement (%) (mean ± SEM) (95% CI)	-14.7 ± 2.2% (-19.29 – -10.18) n= 21	-15.4 ± 3.7% (-23.11 – -7.58) n=21	-20.2 ± 5.4% (-33.01 – -7.38) n=8	-25.0 ± 4.6 (-35.89 – -14.07) n=8	0.48 DOM<br><br>0.17 ND
Hip Flexion- 1st Repetition z-score	-1.8 ± 2.0 (-2.58 – -0.96) n=25	-2.0 ± 1.6 (-2.68 – -1.30) n= 24	-2.3 ±3.2 (-4.63 – 0.00) n=10	-2.0 ± 1.9 (-3.46 – -0.53) n=9	
Hip Flexion- 6th Repetition z-score	-2.2 ± 1.8 (-3.09 – -1.36) n=19	-2.6 ± 2.3 (-3.76 – -1.47) n=18	-2.7 ± 4.1 (-6.12 – 0.67) n=8	-2.4 ± 2.4 (-4.43 – -0.34) n= 8)	
Negative Percent Decrement (%) (mean ± SEM) (95% CI)	-10.9 ± 4.4% (-20.16 – -1.57) n= 19	-11.6 ± 4.8% (-21.75 – -1.35) n= 18	2.7 ± 7.0% (-13.88 – 19.21) n=8	-7.6 ± 5.1% (-19.59 – 4.35) n=8	0.073 DOM<br><br>0.54 ND

**Healthy Volunteers (n=25)**			
**Dynamometry Repetitions** mean z-scores ± SD (95% CI)	**Dominant**	**Non- Dominant**	**t-test p-values[d]** (Healthy Volunteers vs Definite MM)
Elbow Flexion- 1st Repetition z-score	0.3 ± 1.5 (-0.37 – 0.86) n=24	-0.5 ± 1.5 (-1.12 – 0.12) n=25	
Elbow Flexion- 6th Repetition z-score	-0.4 ± 1.6 (-1.04 – 0.35)	-0.9 ± 1.3 (-1.47 - -0.36)	

	n=24	n=25	
Negative Percent Decrement (%)<br>(mean ± SEM) (95% CI)	-6.5 ± 2.8%<br>(-12.34 - -0.69)<br>n=24	-7.0 ± 3.3%<br>(-13.68 - -0.27)<br>n=25	0.036<br>DOM<br><br>0.09<br>ND
Hip Flexion- 1st Repetition z-score	0.4 ± 1.7<br>(-0.37 – 1.07)<br>n=24	0.8 ± 2.6<br>(-0.31 – 1.83)<br>n=25	
Hip Flexion- 6th Repetition z-score	-0.5 ± 1.6<br>(-1.20 – 0.15)<br>n=24	0.25 ± 2.2<br>(-0.64 – 1.14)<br>n=25	
Negative Percent Decrement (%)<br>(mean ± SEM) (95% CI)	-10.0 ± 1.6%<br>(-13.25 - -6.83)<br>n=24	-5.6 ± 1.4%<br>(-8.51 - -2.82)<br>n=25	0.47<br>DOM<br><br>0.23<br>ND
**Healthy Adult Volunteers<br>(n=15)**			
**Dynamometry Repetitions**<br>mean z-scores ± SD (95% CI)	**Dominant**	**Non- Dominant**	**t-test p-values[e]**<br>(Healthy Adult Volunteers vs Adult Definite MM
Elbow Flexion- 1st Repetition z-score	0.5 ± 1.7<br>(-0.45 – 1.38)	-0.57 ± 1.8<br>(-1.58 – 0.43)	
Elbow Flexion- 6th Repetition z-score	-0.3 ± 1.9<br>(-1.33 – 0.82)<br>n=15	-0.88 ± 1.5<br>(-1.76 – 0.00)<br>n=15	
Negative Percent Decrement (%)<br>(mean ± SEM)	-6.5 ± 4.4%<br>(-16.02 – 2.97)<br>n=15	-4.1 ± 4.9%<br>(-14.65 – 6.53)<br>n=15	0.41<br>DOM<br><br>0.32<br>ND

Hip Flexion- 1st Repetition z-score	1.1 ± 1.6<br>(0.17 – 2.03)<br>n=14	2.2 ± 2.4<br>(0.86 – 3.50)<br>n=15	
Hip Flexion- 6th Repetition z-score	-0.1 ± 1.8<br>(-1.11 – 0.91)<br>n=14	1.4 ± 2.0<br>(0.31 – 2.52)<br>n=15	
Negative Percent Decrement (%)<br>(mean ± SEM) (95% CI)	-9.6 ± 2.1%<br>(-14.07 – -5.17)<br>n=14	-7.6 ± 1.9%<br>(-11.70 – -3.54)<br>n=15	0.63<br>DOM<br><br>0.44<br>ND
**Healthy Child Volunteers<br>(n=10)**			
**Dynamometry Repetitions**<br>mean z-scores ± SD (95% CI)	**Dominant**	**Non-Dominant**	**t-test p-values[f]**<br>(Healthy Child Volunteers<br>vs Child Definite MM
Elbow Flexion- 1st Repetition z-score	-0.1 ± 1.0<br>(-0.91 – 0.66)<br>n=9	-0.4 ± 1.0<br>(-1.07 – 0.29)<br>n=10	
Elbow Flexion- 6th Repetition z-score	-0.5 ± 1.1<br>(-1.31 – 0.32)<br>n=9	-1.0 ± 0.9<br>(-1.63 – -0.31)<br>n=10	
Negative Percent Decrement (%)<br>(mean ± SEM) (95% CI)	-6.5 ± 1.9%<br>(-10.92 – -2.05)<br>n=9	-11.4 ± 3.2%<br>(-18.48 – -4.23)<br>n=10	0.034<br>DOM<br><br>0.19<br>ND
Hip Flexion- 1st Repetition z-score	-0.7 ± 1.3<br>(-1.59 – 0.20)<br>n=10	-1.4 ± 0.8<br>(-1.91 – -0.83)<br>n=10	
Hip Flexion- 6th Repetition z-score	-1.1 ± 1.2<br>(-1.97 – -0.25)	-1.5 ± 0.6<br>(-1.96 – -1.04)	

	n=10	n=10	
Negative Percent Decrement (%) (mean ± SEM) (95% CI)	-10.6 ± 2.5% (-16.22 – -5.04) n=10	-2.7 ± 1.6% (-6.38 – 0.92) n=10	0.64 DOM<br><br>0.47 ND
**MM-COAST Composite Score**			
	**Definite (n=53)**	**Unlikely (n=29)**	**t-test *p*-values[c]**
**Composite Score** Mean ± SEM (95% CI)	1.3 ± 0.1 (1.04 – 1.50)	0.5 ± 0.2 (0.14 – 0.84)	0.0005
**Functional Assessment**			
**North Star Scores** mean ± SEM (95% CI)	**Definite (n=58)**	**Unlikely (n=31)**	
	25.1 ± 1.3 (22.61 – 27.63)	29.8 ± 1.1 (27.6 – 31.95)	0.0058

**North Star Ambulatory Assessment scores by item**			
	**Definite MM (n=58)**		
Number (%)	**Score 0 of 2 (unable to achieve)**	**Score 1 of 2 (partially achieves)**	**Score 2 of 2 (achieves fully)**
Item 1: Stand	7 (12.1%)	3 (5.2%)	48 (82.8%)
Item 2: Walk	6 (10.3%)	3 (5.2%)	49 (84.5%)
Item 3: Stand up from chair	2 (3.4%)	7 (12.1/%)	49 (84.5%)
Item 4: Stand on one leg - right	10 (17.2%)	26 (44.8%)	22 (37.9%)
Item 5: Stand on one leg - left	11 (19.0%)	26 (44.8%)	21 (36.2%)
Item 6: Climb box step - right	6 (10.3%)	7 (12.1%)	45 (77.6%)
Item 7: Climb box step - left	7 (12.1%)	8 (12.8%)	43 (74.1%)

Item 8: Descend box - right	6 (10.3%)	12 (20.7%)	40 (69.0%)
Item 9: Descend box - left	8 (13.8%)	8 (13.8%)	42 (72.4%)
Item 10: Gets to sitting	4 (6.9%)	7 (12.1%)	47 (81.0%)
Item 11: Rise from floor	14 (24.1%)	10 (17.2%)	34 (58.6%)
Item 12: Lifts head (n=57)[a]	2 (3.5%)	6 (10.5%)	49 (86.0%)
Item 13: Stands on heels (n=57)[a]	14 (24.6%)	13 (22.8%)	30 (52.6%)
Item 14: Jump (n=57)[a]	11 (19.3%)	2 (3.5%)	44 (77.2%)
Item 15: Hop right leg (n=57)[a]	15 (26.3%)	8 (14.0%)	34 (59.6%)
Item 16: Hop left leg (n=57)[a]	16 (28.1%)	9 (15.8%)	32 (56.1%)
Item: 17 – Run (n=52)[b]	24 (46.2%)	12 (23.1%)	16 (30.8%)

[a] Unable to be tested in 1 participant (n=57)

[b] Unable to be tested in 6 participants (n=52)

[c] t-test comparison of Definite MM to Unlikely Participants

[d] t-test comparison of Definite MM to Healthy Volunteers (n=25)

[e] t-test comparison of Definite Adult MM to Healthy Adult Volunteers (n=15)

[f] t-test comparison of Definite Child MM to Healthy Child Volunteers (n=10)

Table S11. Pearson's Correlations				
**Balance Testing**				
**Correlation to Muscle Strength and Fine Motor Dexterity Tests**		**Number (n)**	**Pearson's r-value**	***p*-value**
Tandem Stance Eyes Closed	Dominant Knee Flexion	21	0.64	0.002
	Dominant Ankle Dorsiflexion	36	0.30	0.072
	Dominant FDT	39	0.33	0.041
	Dominant 9HPT	43	0.32	0.034
Tandem Stance Eyes Open	Dominant Knee Flexion	22	0.004	0.98
	Dominant Ankle Dorsiflexion	37	0.60	<0.0001
	Dominant FDT	39	0.45	0.004
	Dominant 9HPT	43	0.24	0.12
Single Leg Stance Eyes Closed	Dominant Knee Flexion	22	0.48	0.024
	Dominant Ankle Dorsiflexion	37	0.29	0.078
**Fine Motor Dexterity**				
**Correlation to Muscle Strength, 30 second Sit to Stand (30s STS) and 6-Minute Walk Test (6MWT)**		**Number (n)**	**Pearson's r-value**	***p*-value**
Dominant FDT	Dominant Gross Grasp	32	0.10	0.57
	Dominant 3-point Pinch	18	0.27	0.28
	30s STS	25	0.31	0.13
	6MWT z-score	38	0.36	0.026
	6MWT Minute Distance (slope)	38	0.33	0.044

Dominant 9HPT	Dominant Gross Grasp	40	0.33	0.04
	Dominant 3-point Pinch	25	0.22	0.30
	30s STS	28	0.13	0.52
	6MWT z-score	40	0.40	0.011
	6MWT Minute Distance (slope)	40	0.32	0.043
**Exercise Intolerance 30 second Sit to Stand (30s STS)**				
**Correlation to Muscle Strength, Balance and Hand Dexterity Tests**		**Number (n)**	**Pearson's r-value**	***p*-value**
Dominant Hip Flexion		22	0.03	0.91
Dominant Hip Abduction		25	0.08	0.70
Dominant Elbow Flexion		24	-0.01	0.95
Dominant Knee Extension[b]		14	0.02	0.94
Tandem Stance Eyes Open		25	0.12	0.58
Tandem Stance Eyes Closed		25	0.44	0.03
**Muscle Fatigue (Dynamometry Repetitions[d])**				
**Correlation to muscle strength and 30 second Sit to Stand (30s STS)**		**Number (n)**	**Pearson's r-value**	***p*-value**
Dominant Hip Flexion		19	-0.28	0.25
Nondominant Hip Flexion		18	-0.062	0.81
Dominant Elbow Flexion		21	-0.13	0.59
Nondominant Elbow Flexion		21	-0.27	0.24
30s STS[a]	Dominant Hip Flexion	16	0.003	0.99
	Dominant Elbow Flexion	18	-0.02	0.95
**Correlation between Dynamometry Repetitions and 6-Minute Walk Test (6MWT)[a]**		**Number (n)**	**Pearson's r-value**	***p*-value**

Dominant Hip Flexion	6MWT Total Distance	9	0.26	0.50
	6MWT z-score	14	-0.02	0.94
	6MWT Minute Distance (slope)	9	0.01	0.97
Dominant Elbow Flexion	6MWT Total Distance	11	0.31	0.36
	6MWT z-score	16	-0.14	0.61
	6MWT Minute Distance	11	0.12	0.72
**6-Minute Walk Test (6MWT)**		**Number (n)**	**Pearson's r-value**	***p*-value**
**Correlation to muscle strength, 30 second Sit to Stand (30s STS) and Balance Assessments**				
6MWT Total Distance	Dominant Hip flexion	34	0.31	0.08
	Dominant Ankle Dorsiflexion	35	0.31	0.071
	Dominant Elbow Flexion	36	0.24	0.16
	Dominant Wrist Extension	33	0.26	0.14
	30s STS	23	0.33	0.13
6MWT z-scores	Dominant Hip flexion	37	0.20	0.23
	Dominant Ankle Dorsiflexion	39	0.67	<0.0001
	Dominant Elbow Flexion	37	0.36	0.030
	Dominant Wrist Extension	38	0.31	0.055
	30s STS	30	0.80	<0.0001
	Tandem Stance Eyes Open	40	0.34	0.034
	Tandem Stance Eyes Closed	39	0.47	0.002
6MWT Minute Distance (slope)	Dominant Hip flexion	34	0.29	0.095
	Dominant Ankle Dorsiflexion	35	0.41	0.013
	Dominant Elbow Flexion	36	0.26	0.12
	Dominant Wrist Extension	33	0.32	0.066
	30s STS	23	0.34	0.12

	Tandem Stance Eyes Open	40	0.37	0.017
	Tandem Stance Eyes Closed	39	0.33	0.037
**Functional Assessments**				
**Correlation to North Star Ambulatory Assessment**		**Number (n)**	**Pearson's r-value**	***p*-value**
Balance	Tandem Stance Eyes Open	44	0.54	<0.0001
	Tandem Stance Eyes Closed	43	0.23	0.14
	Single Leg Stance Eyes Closed	44	0.20	0.21
30 second Sit to Stand (30s STS)		27	0.48	0.012
Fine Motor Dexterity	Dominant 9HPT	46	0.56	<0.0001
	Dominant FDT	39	0.50	0.001
Muscle Strength	Dominant Wrist Extension	45	0.41	0.005
	Dominant Pinch	29	0.37	0.051
	Dominant Hip Flexion	46	0.33	0.028
	Dominant Hip Abduction	48	0.37	0.009
	Dominant Ankle Dorsiflexion	45	0.54	<0.0001
	Dominant Knee Flexion[b]	29	0.48	0.008
	Dominant Knee Extension[b]	29	0.64	0.0002
	Dominant Elbow Flexion	50	0.25	0.075
	Dominant Elbow Extension	48	0.08	0.58
	Dominant Shoulder Abduction	48	0.24	0.095
	Dominant Shoulder External Rotation[c]	18	-0.01	0.98
	Neck Flexion	31	0.07	0.71
Muscle Fatigue dynamometry repetitions	Dominant Hip Flexion	15	-0.23	0.41
	Dominant Elbow Flexion	17	-0.01	0.96

6MWT	Total Distance	40	0.67	<0.0001
	z-score	44	0.55	<0.0001
	Minute Distance (slope)	40	0.68	<0.0001
**Correlation to MM-COAST Composite Score**		**Number (n)**	**Pearson's r-value**	***p*-value**
Balance	Tandem Stance Eyes Open	38	-0.61	<0.0001
	Tandem Stance Eyes Closed	37	-0.63	<0.0001
30 second Sit to Stand (30s STS)		25	-0.58	0.002
Fine Motor Dexterity	Dominant 9HPT	41	-0.62	<0.0001
	Dominant FDT	34	-0.70	<0.0001
Muscle Strength	Dominant Wrist Extension	47	-0.50	<0.0001
	Dominant Hip Flexion	47	-0.50	<0.0001
	Dominant Ankle Dorsiflexion	47	-0.60	<0.0001
	Dominant Elbow Flexion	52	-0.49	<0.0001
Muscle Fatigue dynamometry repetitions	Dominant Hip Flexion	15	0.12	0.68
	Dominant Elbow Flexion	17	-0.13	0.62
6MWT	Total Distance	37	-0.68	<0.0001
	z-score	42	-0.72	<0.0001
	Minute Distance (slope)	37	-0.71	<0.0001
North Star Ambulatory Assessment	Total Score	52	-0.64	<0.0001

[a] Analysis with negative percent decrement of dynamometry repetitions testing
[b] Testing performed in Child MM group, not tested in adult cohort per protocol [5]
[c] Testing performed in Adult MM group, not tested in child cohort per protocol [10]
[d] Correlation to negative percent decrement values

Table S12. Summary of data that led to final selection of MM-COAST assessments

Domains	Tests	Background Literature and Relevant results	Rationale for inclusion in MM-COAST
Muscle Strength	Dynamometry of Upper and Lower Extremities	• Symmetric pattern of muscle weakness occurs in MM, p<0.0001 (Figure S1).<br><br>• Principal component analysis (PCA) revealed all muscle groups, except for neck flexion and pinch, correlate to each other (Figure S2).<br><br>• Strength in most muscle groups correlates positively with age, height, and weight (p<0.05) after controlling for sex, including elbow flexion, wrist extension, hip flexion, and ankle dorsiflexion (Table S9).<br><br>• Hip flexion and elbow flexion were the weakest proximal muscles and ankle dorsiflexion and wrist extension were the weakest distal muscles in MM cohort-tested in both children and adults (Table S6, Figure 2a and 2d). These muscles are involved in daily activities such as lifting objects, typing, ambulation, and walking up the stairs, which are essential activities of daily living.<br><br>• **Relevant significant correlations between dominant side muscle strength with North Star Ambulatory Assessment (NSAA) and other domain assessments as follows:**	• Recommended dominant side testing for the MM-COAST.<br><br>• Recommended one proximal and one distal muscle group, each in upper and lower extremities only.<br><br>• As normative data is based on age and sex, the weakest muscles in the MM group (elbow flexion, wrist extension, hip flexion, and ankle dorsiflexion) were selected to represent muscle strength across all muscle groups.<br><br>• Hip flexion, elbow flexion, ankle dorsiflexion and wrist extension were selected as the target muscles to be assessed in MM-COAST<br><br>• Muscle strength measured by dynamometry is included in MM-COAST due to significant correlations between ankle dorsiflexion strength with 6-minute walk test slope and z-score, elbow flexion

		Ankle dorsiflexion strength with 6-minute walk test minute distance slope (r=0.41, p=0.013, n=35) and 6-minute walk test z-scores (r=0.67, p<0.0001, n=39) (r=0.60, p<0.0001, n=39).<br><br>Elbow flexion strength with 6-minute walk test z-score (r=0.36, p=0.03, n=37)<br><br>Ankle dorsiflexion strength with Tandem Stance Eyes Open (r=0.60, p<0.0001, n=37)<br><br>Ankle dorsiflexion strength with NSAA score (r=0.54, p<0.0001, n=45).<br><br>Hip flexion strength with NSAA score (r=0.33, p=0.03, n=46)<br><br>Wrist extension strength with NSAA score (r=0.41, p=0.005, n=45)<br><br>• Non-ambulatory participants (4/6) were able to complete dynamometry assessments in the muscle strength domain to contribute towards an MM-COAST composite score.	strength with 6-minute walk z-score; ankle dorsiflexion strength with balance assessment; and ankle dorsiflexion, hip flexion, and wrist extension strength correlations to NSAA score, which indicates clinical meaning.<br><br>• Non-ambulatory individuals can be included in MM-COAST assessments.
Muscle Fatigue	Dynamometry repetitions at elbow flexion	• Symmetric pattern of muscle weakness occurs in MM (Figure S1, p<0.0001).<br><br>• Elbow flexion was one of the weakest proximal muscles in MM cohort -tested in	• Recommended dominant side testing for the MM-COAST.<br><br>• Elbow flexors selected as target muscle to be tested for dynamometry repetitions

		both adults and children (Table S6, Figures 2a and 2d).<br><br>• Of the 4 weakest muscle groups in the MM cohort, elbow flexors have been shown to be the most reliable muscle group for hand held dynamometry (ICC .934, (95%CI 0.814-0.958) in adults [10] and is one of the more reliable muscle groups to be tested in children as young as 4 and 5 years old [5].<br><br>• Elbow flexion repetitions were observed to be more reliably and consistently performed across all age groups in the MM cohort for elbow flexion percent decrement (-15.9% in child, -12.7% in adult) compared to hip flexion percent decrement (-14.0% in child, -5.5% in adult).<br><br>• **Relevant non-significant correlations observed in definite MM cohort:**<br><br>Dynamometry repetitions percent decrement at dominant hip flexion with dominant hip flexion muscle strength (r=-0.28, p=0.25, n=19).<br><br>Dynamometry repetitions percent decrement at dominant elbow flexion with dominant elbow flexion muscle strength (r=-0.13, p=0.58, n=21).	assessment of muscle fatigue as it is a weak muscle group in MM and has been shown to be one of the most reliable muscle groups for testing using hand-held dynamometry across age groups [10].<br><br>• Hip flexion dynamometry repetition assessment was not included in MM-COAST because feasibility across all ages may be a barrier (See Table S4).<br><br>• Dynamometry repetitions assessment of muscle fatigue is included in the MM-COAST as dynamometry repetitions is distinct to dynamometry muscle strength assessment.

		Dynamometry repetitions percent decrement with 30 second sit to stand test (dominant hip flexion: r=0.003, p=0.99, n=16 and dominant elbow flexion: r=-0.02, p=0.95, n=18).<br><br>Dynamometry repetitions percent decrement on with 6-minute walk test minute distance slope (dominant hip flexion: r=0.02, p=0.97, n=9 and dominant elbow flexion: r=0.12, p=0.72, n=11).	• Dynamometry repetitions assessment of muscle fatigue is included in the MM-COAST as dynamometry repetitions is distinct to exercise intolerance (30 second sit to stand and 6-minute walk test) assessments.
Balance	Single Leg Stance Eyes Closed<br><br>Tandem Stance Eyes Open and Eyes Closed	• Recognized high prevalence of imbalance as >70 percent of people surveyed in prior study reported imbalance [35].<br><br>• **Relevant significant correlations observed in definite MM cohort:**<br><br>Tandem Stance Eyes Open with dominant ankle dorsiflexion strength (r=0.60, p<0.0001, n=37)<br><br>Tandem Stance Eyes Open with 6-minute walk test minute distance slope (r=0.37, p=0.017, n=40) and 6-minute walk test z-scores (r=0.34, p=0.034, n=40).<br><br>Tandem Stance Eyes Open with dominant Functional Dexterity Test (r=0.45, p=0.004, n=39).<br><br>Tandem Stance Eyes Open with NSAA scores (r=0.54, p<0.0001, n=44).	• Determined that MM-COAST should include balance assessments<br><br>• Balance assessments included in MM-COAST due to significant correlation with ankle dorsiflexion and knee flexion muscle strength, dexterity, 6-minute walk test minute distance slope and z-scores, and to the NSAA score, which indicates clinical meaning.

		Tandem Stance Eyes Closed with dominant knee flexion strength (r=0.64, p=0.002, n=21).<br><br>Tandem Stance Eyes Closed with 6-minute walk test minute distance slope (r=0.33, p=0.037, n=39), and 6-minute walk test z-score (r=0.47, p=0.002, n=39).<br><br>Tandem Stance Eyes Closed with dominant Functional Dexterity Test scores (r=0.33, p=0.041, n=39).<br><br>Tandem Stance Eyes Closed with dominant Nine Hole Peg Test scores (r=0.32, p=0.034, n=43).	
Exercise intolerance	30 second Sit to Stand (30s STS)	• 30s STS is a standard measure of exercise intolerance and measures ability to rise from a chair, an activity that occurs repeatedly throughout the day [36].<br><br>• **Relevant significant correlations observed in definite MM cohort:**<br><br>30s STS with Tandem Stance Eyes Closed (r=0.44, p=0.03 n=25).<br><br>30s STS with 6-minute walk test z-scores (r=0.80, p<0.0001, n=30).	• 30s STS is included in MM-COAST as it is a strength-based measurement of exercise intolerance [37, 38].<br><br>• Included in MM-COAST due to significant correlations with balance, 6MWT z-scores, and the NSAA score, which indicates clinical meaning.

		30s STS with NSAA scores (r=0.48, p=0.01, n=27).	
Exercise intolerance	6-Minute Walk Test (6MWT)	• 6MWT is a mobility-based assessment of exercise intolerance.<br><br>• **Relevant significant correlations observed in definite MM cohort:**<br><br><u>6MWT z-score:</u><br>6MWT z-scores with dominant ankle dorsiflexion strength (r=0.67, p<0.0001, n= 39).<br><br>6MWT z-scores with dominant elbow flexion strength (r=0.36, p=0.03, n=37).<br><br>6MWT z-scores with Tandem Stance Eyes Closed (r=0.47, p=0.002, n=39).<br><br>6MWT z-scores with 30s STS (r=0.80, p<0.0001, n=30).<br><br>6MWT z-scores with dominant Nine Hole Peg Test (r=0.40, p=0.011, n= 40).<br><br>6MWT z-scores with dominant Functional Dexterity Test (r=0.36, p=0.03, n= 38).<br><br>6MWT z-scores with NSAA score: (r=0.55, p<0.0001, n=44).	• 6MWT z-scores was included in the MM-COAST due to correlations with muscle strength, balance assessments, 30s STS, and dexterity assessments as well as NSAA, which indicates clinical meaning.

		6MWT total distance walked with NSAA score: (r=0.672, p<0.0001, n=40).<br><br>• **Relevant significant correlations observed in definite MM cohort:**<br><br><u>6MWT minute distance slope:</u><br>6MWT minute distance slope with dominant ankle dorsiflexion strength (r=0.41, p=0.013, n=35).<br><br>6MWT minute distance slope with dominant Nine Hole Peg Test (r=0.32, p=0.04, n=40).<br><br>6MWT minute distance slope with dominant Functional Dexterity Test (r=0.33, p=0.04, n=38).<br><br>6MWT minute distance slope with Tandem Stance Eyes Open (r=0.37, p=0.017, n=40).<br><br>6MWT minute distance slope with Tandem Stance Eyes Closed (r=0.33, p=0.037, n=39).<br><br>6MWT minute distance slope with NSAA score (r=0.68, p=<0.0001, n=40).<br><br>6MWT minute distance slope with MM-COAST Composite score (r=-0.69, p<0.0001, n=42).	• 6MWT minute distance slope analysis is considered meaningful due to significant correlations with ankle dorsiflexion strength, dexterity, balance, and NSAA score, as well the MM-COAST composite score.<br><br>• The 6MWT minute distance slope was not correlated to 30s STS (r=0.34, p=0.12, n=23), which suggests that 30s STS and 6MWT minute distance assessments are distinct measures of exercise intolerance, and therefore both assessments of exercise intolerance were included in the MM-COAST.

Dexterity	Nine Hole Peg Test (9 HPT)	• **Relevant significant correlations observed in definite MM cohort between dominant side 9HPT and other domain assessments:**<br><br>9HPT with dominant gross grasp (r=0.33, p=0.04, n=40), but not with pinch strength (r=0.22, p=0.30, n=25).<br><br>9HPT with Tandem Stance Eyes Closed (r=0.32, p=0.03, n=43).<br><br>9HPT with minute distance slope (r=0.32, p=0.04, n=40) and 6MWT z-scores (r=0.40, p=0.011, n= 40).<br><br>9HPT with NSAA (r=0.56, p<0.0001, n=46).	• 9HPT is included in MM-COAST due to significant correlations to gross grasp, balance measures, 6MWT minute distance slope and z-scores, and NSAA score, which indicates clinical meaning.
Dexterity	Functional Dexterity Test (FDT)	• **Relevant significant correlations observed in definite MM cohort between dominant side FDT and other domain assessments:**<br><br>FDT with both Tandem Stance Eyes Open (r=0.45, p=0.004, n=39) and Tandem Stance Eyes Closed (r=0.33, p=0.04, n=39).<br><br>FDT with 6MWT minute distance slope (r=0.33, p=0.04, n=38) and 6MWT z-scores (r=0.36, p=0.03, n= 38).	• FDT is included in MM-COAST due to correlations to balance measures, 6MWT minute distance slope and z-scores, and NSAA score, which indicates clinical meaning.

		FDT with NSAA total score (r=0.50, p=0.001, n=39).	
MM - COAST Composite Score		• **Relevant significant correlations observed in definite MM cohort:**<br><br>MM-COAST Composite Score with dominant hip flexion strength (r=-0.50, p<0.0001, n=47).<br><br>MM-COAST Composite Score with dominant elbow flexion strength (r=-0.49, p<0.0001, n=52).<br><br>MM-COAST Composite Score with dominant ankle dorsiflexion strength (r=-0.6, p<0.0001, n=47).<br><br>MM-COAST Composite Score with dominant wrist extension strength (r=-0.5, p<0.0001, n=47).<br><br>MM-COAST Composite Score with Tandem Stance Eyes Open (r=-0.61, p<0.0001, n=38).<br><br>MM-COAST Composite Score with Tandem Stance Eyes Closed (r=-0.63, p<0.0001, n=37).	• Results demonstrate clinical meaning of the MM-COAST Composite Score as it correlated with all MM-COAST domains except muscle fatigue, which is likely due to the small sample size in our dynamometry muscle fatigue repetitions assessment.

		MM-COAST Composite Score with dominant FDT (r=-0.7, p<0.0001, n=34).<br><br>MM-COAST Composite Score with dominant 9HPT (r=-0.62, p<0.0001, n=41).<br><br>MM-COAST Composite Score with 30s STS (r=-0.58, p=0.002, n=25).<br><br>MM-COAST Composite Score with 6MWT total distance walked (r=-0.68, p<0.0001, n=37).<br><br>MM-COAST Composite Score with 6MWT minute distance slope (r=-0.71, p<0.0001, n=37).<br><br>MM-COAST Composite Score with 6MWT z-scores (r=-0.72, p<0.0001, n=42).<br><br>MM-COAST Composite Score with NSAA (r=-0.64, p<0.0001, n=52).	

©2020 The Children's Hospital of Philadelphia. All rights reserved

References

1. Prasun, P. and D.D. Koeberl, *Mitochondrial neurogastrointestinal encephalomyopathy (MNGIE)-like phenotype in a patient with a novel heterozygous POLG mutation.* J Neurol, 2014. **261**(9): p. 1818-9.

2. Miyake, N., et al., *X-linked hypomyelination with spondylometaphyseal dysplasia (H-SMD) associated with mutations in AIFM1.* Neurogenetics, 2017. **18**(4): p. 185-194.

3. Gustafson, M.A., et al., *Mitochondrial single-stranded DNA binding protein novel de novo SSBP1 mutation in a child with single large-scale mtDNA deletion (SLSMD) clinically manifesting as Pearson, Kearns-Sayre, and Leigh syndromes.* PLoS One, 2019. **14**(9): p. e0221829.

4. Mathiowetz, V., D.M. Wiemer, and S.M. Federman, *Grip and pinch strength: norms for 6- to 19-year-olds.* Am J Occup Ther, 1986. **40**(10): p. 705-11.

5. Beenakker, E.A., et al., *Reference values of maximum isometric muscle force obtained in 270 children aged 4-16 years by hand-held dynamometry.* Neuromuscul Disord, 2001. **11**(5): p. 441-6.

6. Mathiowetz, V., et al., *Grip and pinch strength: normative data for adults.* Arch Phys Med Rehabil, 1985. **66**(2): p. 69-74.

7. Merlini, L., et al., *Reliability of hand-held dynamometry in spinal muscular atrophy.* Muscle Nerve, 2002. **26**(1): p. 64-70.

8. Solari, A., et al., *Reliability of clinical outcome measures in Charcot-Marie-Tooth disease.* Neuromuscul Disord, 2008. **18**(1): p. 19-26.

9. Mathiowetz, V., et al., *Reliability and validity of grip and pinch strength evaluations.* J Hand Surg Am, 1984. **9**(2): p. 222-6.

10. Phillips, B.A., S.K. Lo, and F.L. Mastaglia, *Muscle force measured using "break" testing with a hand-held myometer in normal subjects aged 20 to 69 years.* Arch Phys Med Rehabil, 2000. **81**(5): p. 653-61.

11. Kim, S.-G. and Y.-S. Lee, *The intra- and inter-rater reliabilities of lower extremity muscle strength assessment of healthy adults using a hand held dynamometer.* Journal of Physical Therapy Science, 2015. **27**(6): p. 1799-1801.

12. Roy, J.-S.P.T.P., et al., *The Concurrent Validity of a Hand-held versus a Stationary Dynamometer in Testing Isometric Shoulder Strength.* Journal of hand therapy, 2009. **22**(4): p. 320-327.

13. Stuberg, W.A. and W.K. Metcalf, *Reliability of quantitative muscle testing in healthy children and in children with Duchenne muscular dystrophy using a hand-held dynamometer.* Phys Ther, 1988. **68**(6): p. 977-82.

14. Mazzone, E.S., et al., *Reliability of the North Star Ambulatory Assessment in a multicentric setting.* Neuromuscul Disord, 2009. **19**(7): p. 458-61.

15. Scott, E., et al., *Development of a functional assessment scale for ambulatory boys with Duchenne muscular dystrophy.* Physiother Res Int, 2012. **17**(2): p. 101-9.

16. McKay, M.J., et al., *Reference values for developing responsive functional outcome measures across the lifespan.* Neurology, 2017. **88**(16): p. 1512-1519.

17. Wuang, Y.P. and C.Y. Su, *Reliability and responsiveness of the Bruininks-Oseretsky Test of Motor Proficiency-Second Edition in children with intellectual disability.* Res Dev Disabil, 2009. **30**(5): p. 847-55.

18. Eden, M.M., J. Tompkins, and J.L. Verheijde, *Reliability and a correlational analysis of the 6MWT, ten-meter walk test, thirty second sit to stand, and the linear analog scale of function in patients with head and neck cancer.* Physiother Theory Pract, 2018. **34**(3): p. 202-211.

19. Petersen, C., et al., *Reliability and Minimal Detectable Change for Sit-to-Stand Tests and the Functional Gait Assessment for Individuals With Parkinson Disease.* J Geriatr Phys Ther, 2017. **40**(4): p. 223-226.

20. Smith, Y.A., E. Hong, and C. Presson, *Normative and validation studies of the Nine-hole Peg Test with children.* Percept Mot Skills, 2000. **90**(3 Pt 1): p. 823-43.

21. Feys, P., et al., *The Nine-Hole Peg Test as a manual dexterity performance measure for multiple sclerosis.* Mult Scler, 2017. **23**(5): p. 711-720.

22. Sartorio, F., et al., *The Functional Dexterity Test: test-retest reliability analysis and up-to date reference norms.* J Hand Ther, 2013. **26**(1): p. 62-7; quiz 68.

23. Tissue, C.M., et al., *Validity and reliability of the Functional Dexterity Test in children.* J Hand Ther, 2017. **30**(4): p. 500-506.

24. Montes, J., et al., *Six-Minute Walk Test demonstrates motor fatigue in spinal muscular atrophy.* Neurology, 2010. **74**(10): p. 833-8.

25. Ries, J.D., et al., *Test-retest reliability and minimal detectable change scores for the timed "up & go" test, the six-minute walk test, and gait speed in people with Alzheimer disease.* Phys Ther, 2009. **89**(6): p. 569-79.

26. Li, A.M., et al., *The six-minute walk test in healthy children: reliability and validity.* Eur Respir J, 2005. **25**(6): p. 1057-60.

27. Mylius, C.F., D. Paap, and T. Takken, *Reference value for the 6-minute walk test in children and adolescents: a systematic review.* Expert Rev Respir Med, 2016. **10**(12): p. 1335-1352.

28. McKay, M.J., et al., *1000 Norms Project: protocol of a cross-sectional study cataloging human variation.* Physiotherapy, 2016. **102**(1): p. 50-6.

29. McKay, M.J., et al., *Normative reference values for strength and flexibility of 1,000 children and adults.* Neurology, 2017. **88**(1): p. 36-43.

30. van der Ploeg, R.J., V. Fidler, and H.J. Oosterhuis, *Hand-held myometry: reference values.* J Neurol Neurosurg Psychiatry, 1991. **54**(3): p. 244-7.

31. Aaron, D.H. and C.W. Jansen, *Development of the Functional Dexterity Test (FDT): construction, validity, reliability, and normative data.* J Hand Ther, 2003. **16**(1): p. 12-21.

32. Gogola, G.R., et al., *Hand dexterity in children: administration and normative values of the functional dexterity test.* J Hand Surg Am, 2013. **38**(12): p. 2426-31.

33. Lee-Valkov, P.M., et al., *Measuring normal hand dexterity values in normal 3-, 4-, and 5-year-old children and their relationship with grip and pinch strength.* J Hand Ther, 2003. **16**(1): p. 22-8.

34. Duff, S.V., et al., *Innovative evaluation of dexterity in pediatrics.* J Hand Ther, 2015. **28**(2): p. 144-9; quiz 150.

35. Zolkipli-Cunningham, Z., et al., *Mitochondrial disease patient motivations and barriers to participate in clinical trials.* PLoS One, 2018. **13**(5): p. e0197513.

36. Roldan-Jimenez, C., P. Bennett, and A.I. Cuesta-Vargas, *Muscular Activity and Fatigue in Lower-Limb and Trunk Muscles during Different Sit-To-Stand Tests.* PLoS One, 2015. **10**(10): p. e0141675.

37. Crockett, K., et al., *The Relationship of Knee-Extensor Strength and Rate of Torque Development to Sit-to-Stand Performance in Older Adults.* Physiother Can, 2013. **65**(3): p. 229-35.

38. Roldan Jimenez, C., et al., *Fatigue Detection during Sit-To-Stand Test Based on Surface Electromyography and Acceleration: A Case Study.* Sensors (Basel), 2019. **19**(19).

Supporting Information Captions

Fig. S1 Dynamometry muscle strength testing revealed a symmetric pattern of muscle weakness in MM. Pearson's correlation of Right: Left was performed. R-values are presented above each muscle group. All muscle groups had significant p-values ($p<0.0001$). Dots represent outliers. Results indicate significant symmetry in each muscle group tested.

Fig. S2 Principle component analysis (PCA) of dynamometry-measured muscle strength. PCA indicates that all muscle groups correlate with one another with the exception of neck flexion and pinch.

Fig. S3 Pearson's correlation between 6-minute walk test (6MWT) total distance walked and age, height, and weight. 3a-c. Scatter plots indicate lack of interaction between total distance walked and age, height and weight. 3d. Two sample t-test comparison showed no difference in the total distance walked between genders.
