# Supplementary figures and images for: Development of a Mitochondrial Myopathy-Composite Assessment Tool
Source: JCSM Clin Rep. Author manuscript; Available in PMC 2022 Jan 21. (PMC8782422)

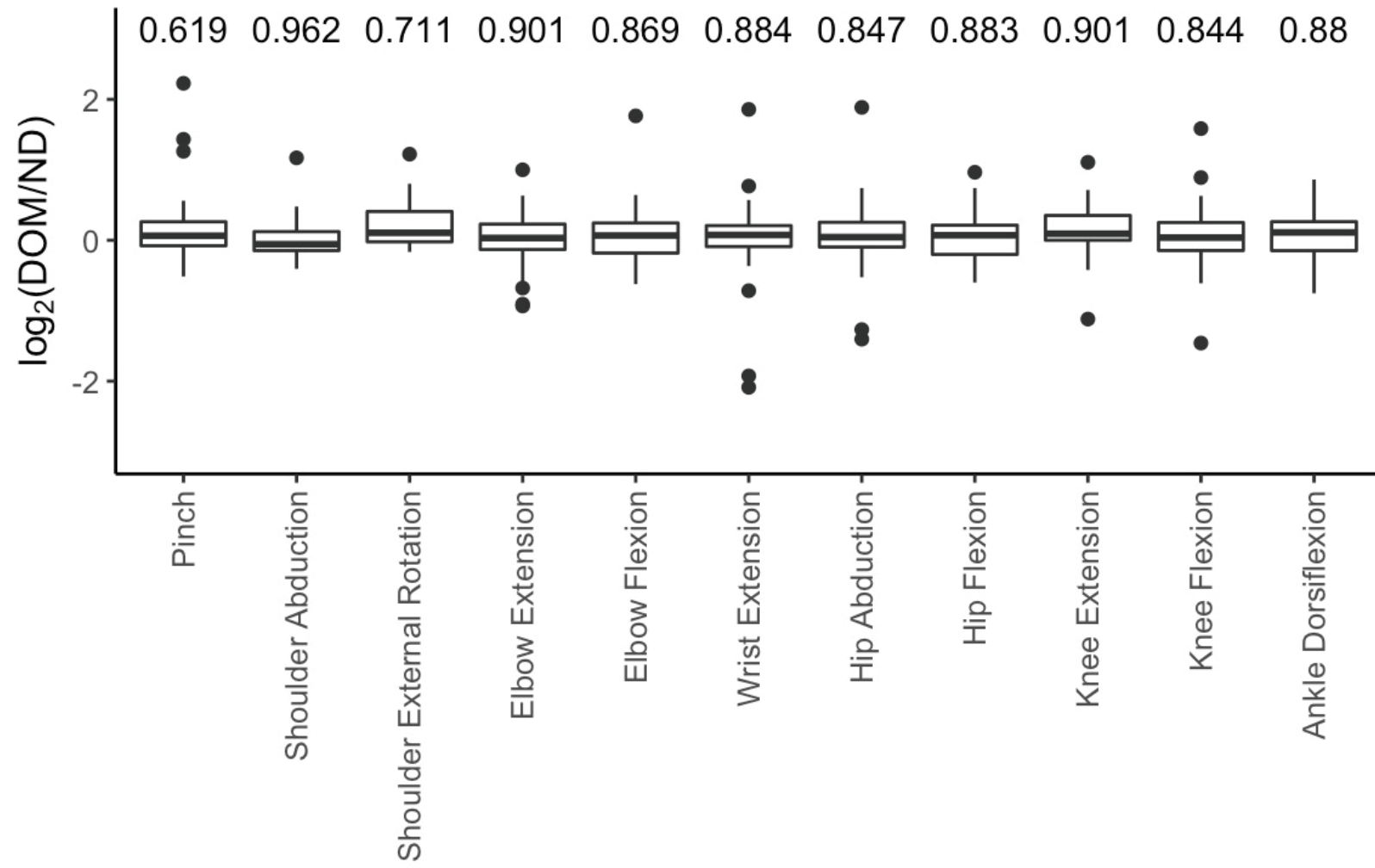

Supplement: Supp Figure 1 — Figure S1. Dynamometry muscle strength testing revealed symmetric pattern of muscle weakness in MM. Pearson’s correlation of Right: Left was performed. R-values are presented above each muscle group. All muscle groups had significant p-values (p < 0.0001). Dots represent outliers. Results indicate significant symmetry in each muscle group tested. [file NIHMS1767033-supplement-Supp_Figure_1.pdf]

1 - Correlation Coefficient

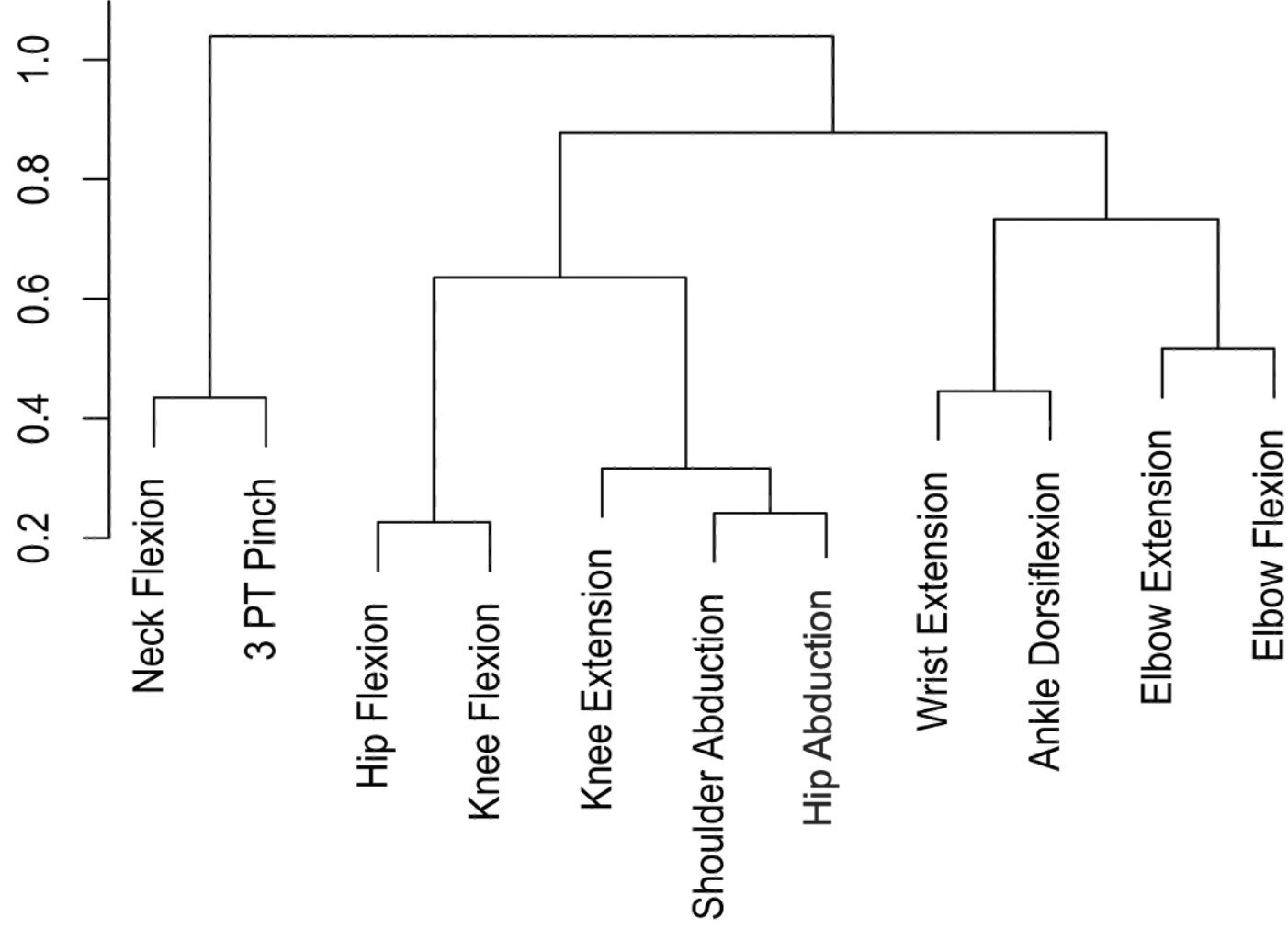

Supplement: Supp Figure 2 — Figure S2. Principle component analysis (PCA) of dynamometry-measured muscle strength. PCA indicates that all muscle groups correlate with one another with the exception of neck flexion and pinch. [file NIHMS1767033-supplement-Supp_Figure_2.pdf]

**a.**

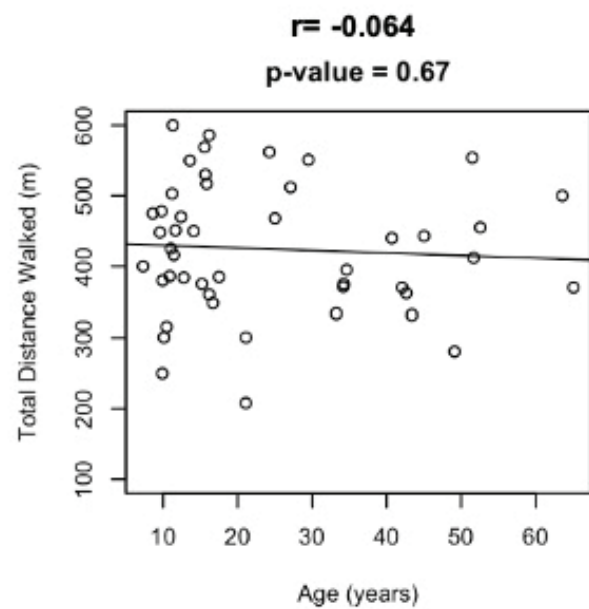

**b.**

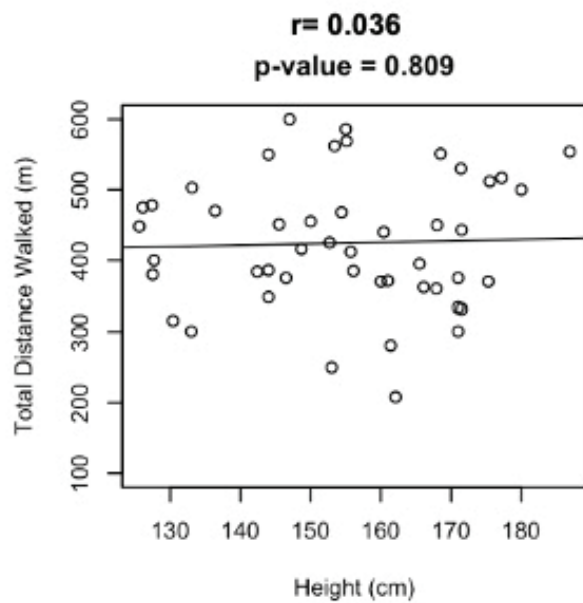

**c.**

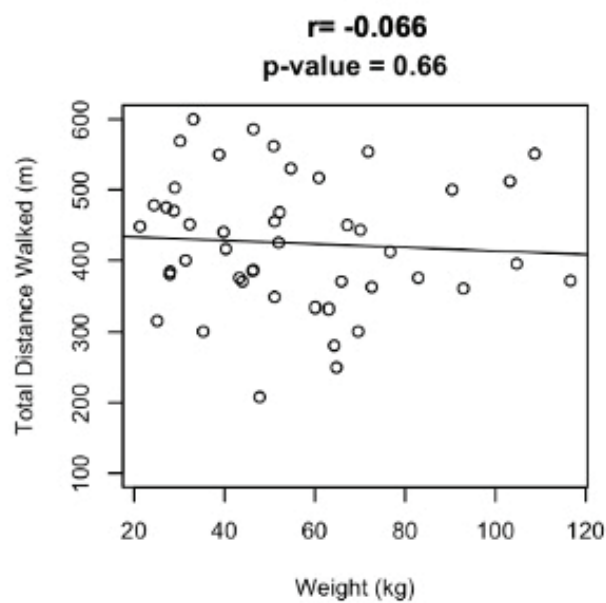

**d.**

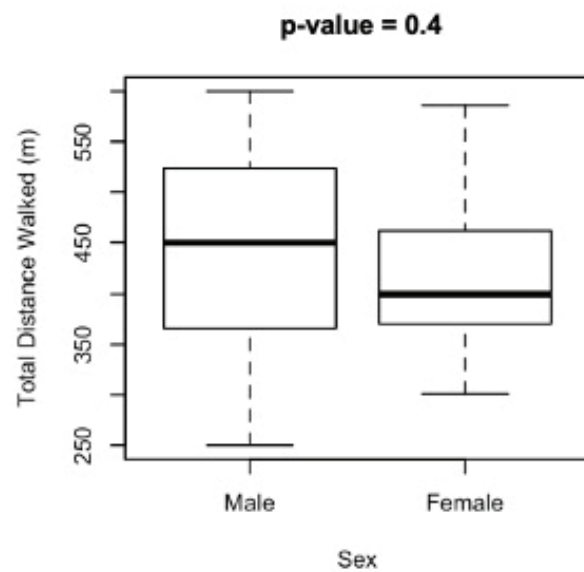

Supplement: Supp Figure 3 — Figure S3. Pearson’s correlation between 6-minute walk test (6MWT) total distance walked and age, height, and weight. 3a-c. Scatter plots indicate lack of interaction between total distance walked and age, height and weight. 3d. Two sample t-test comparison showed no difference in the total distance walked between genders. [file NIHMS1767033-supplement-Supp_Figure_3.pdf]
