# Supplementary material for: Development of a Mitochondrial Myopathy-Composite Assessment Tool
Source: JCSM Clin Rep. Author manuscript; Available in PMC 2022 Jan 21. (PMC8782422)
Supplement: Supp Figure Legends [file NIHMS1767033-supplement-Supp_Figure_Legends.pdf]

Supplemental Figure Legends

Figure S1. Dynamometry muscle strength testing revealed symmetric pattern of muscle weakness in MM. Pearson's correlation of Right: Left was performed. R-values are presented above each muscle group. All muscle groups had significant p-values ($p < 0.0001$). Dots represent outliers. Results indicate significant symmetry in each muscle group tested.

Figure S2. Principle component analysis (PCA) of dynamometry-measured muscle strength. PCA indicates that all muscle groups correlate with one another with the exception of neck flexion and pinch.

Figure S3. Pearson's correlation between 6-minute walk test (6MWT) total distance walked and age, height, and weight. 3a-c. Scatter plots indicate lack of interaction between total distance walked and age, height and weight. 3d. Two sample t-test comparison showed no difference in the total distance walked between genders.
